# *In-situ* formed elastin-based hydrogels enhance wound healing via promoting innate immune cells recruitment and angiogenesis

**DOI:** 10.1016/j.mtbio.2022.100300

**Published:** 2022-05-21

**Authors:** Duo-Mei Tian, Huan-Huan Wan, Jia-Reng Chen, Yong-Bin Ye, Yong He, Yu Liu, Lu-Yao Tang, Zhong-Yuan He, Kai-Zheng Liu, Chong-Jian Gao, Sheng-Lin Li, Qian Xu, Zheng Yang, Chen Lai, Xiao-Jun Xu, Chang-Shun Ruan, Yun-Sheng Xu, Chao Zhang, Liang Luo, Le-Ping Yan

**Affiliations:** a Department of Critical Care Medicine, The Seventh Affiliated Hospital, Sun Yat-sen University, Shenzhen, 518107, PR China; b Scientific Research Center, The Seventh Affiliated Hospital of Sun Yat-sen University, Shenzhen, 518107, PR China; c Department of Dermatovenereology, The Seventh Affiliated Hospital, Sun Yat-sen University, Shenzhen, 518107, PR China; d School of Biomedical Engineering, Sun Yat-sen University, Shenzhen, 518107, PR China; e Department of Hematology, Zhongshan Hospital of Sun Yat-Sen University and Zhongshan City People's Hospital, Zhongshan, 528403, Guangdong, PR China; f Department of Orthopaedic Surgery, The Seventh Affiliated Hospital, Sun Yat-sen University, Shenzhen, 518107, PR China; g Research Center for Human Tissue and Organs Degeneration, Institute of Biomedicine and Biotechnology, Shenzhen Institute of Advanced Technology, Chinese Academy of Sciences, Shenzhen, 518071, PR China; h Shenzhen Testing Center of Medical Devices, No.28, Gaoxin Central 2nd Avenue, Nanshan District, Shenzhen, 518057, PR China; i The Charles Institute of Dermatology, School of Medicine, University College Dublin, Belfield, Dublin 4, Eircode, D04 V1W8, Ireland; j Department of Pathology, The Seventh Affiliated Hospital, Sun Yat-sen University, Shenzhen, 518107, PR China; k Shenzhen Key Laboratory of Human Tissue Regeneration and Repair, Shenzhen Institute Peking University, Shenzhen, 518057, PR China; l Department of Hematology, The Seventh Affiliated Hospital, Sun Yat-sen University, Shenzhen, 518107, PR China; m Guangdong Provincial Key Laboratory of Digestive Cancer Research, The Seventh Affiliated Hospital, Sun Yat-sen University, Shenzhen, 518107, PR China

**Keywords:** Elastin, Immunomodulatory biomaterials, *In-situ* formed hydrogel, Wound healing, Neutrophils, Macrophages

## Abstract

Harnessing the inflammation and angiogenesis is extremely important in wound healing. In this study, we developed bioactive elastin-based hydrogels which can recruit and modulate the innate immune cells and accelerate angiogenesis in the wound site and subsequently improve wound regeneration. These hydrogels were formed by visible-light cross-linking of acryloyl-(polyethylene glycol)-N-hydroxysuccinimide ester modified elastin with methacrylated gelatin, in order to mimic dermal microenvironment. These hydrogels showed highly tunable mechanical properties, swelling ratios and enzymatic degradation profiles, with moduli within the range of human skin. To mimic the *in vivo* degradation of the elastin by elastase from neutrophils, *in vitro* co-culture of the hydrogels and neutrophils was conducted. The derived conditioned medium containing elastin derived peptides (EDP-conditioned medium) promoted the expression of both M1 and M2 markers in M1 macrophages *in vitro*. Additionally, the EDP-conditioned medium induced superior tube formation of endothelia cells in Matrigel. In mice wound model, these elastin-based hydrogels attracted abundant neutrophils and predominant M2 macrophages to the wound and supported their infiltration into the hydrogels. The outstanding immunomodulatory effect of the elastin-based hydrogels resulted in superior angiogenesis, collagen deposition and dermal regeneration. Hence, these elastin-based hydrogels can be a promising regenerative platform to accelerate wound repair.

## Graphical abstract

Elastin-based hydrogels were synthesized by blue-light crosslinking of modified elastin and GelMA, and then applied in mice dermal wound. During inflammation stage of wound healing, innate immune cells neutrophils and macrophages arrive in the wound site. The neutrophils secret neutrophil elastase to degrade the hydrogels, resulting in accelerated elastin-derived peptides (EDPs) release and facilitating the infiltration of neutrophiles and macrophages into the hydrogels. The proangiogenic factors produced by the innate immune cells and the EDPs work together to promote angiogenesis and tissue growth in the wound, and eventually accelerated wound healing is achieved.

**Figure:**
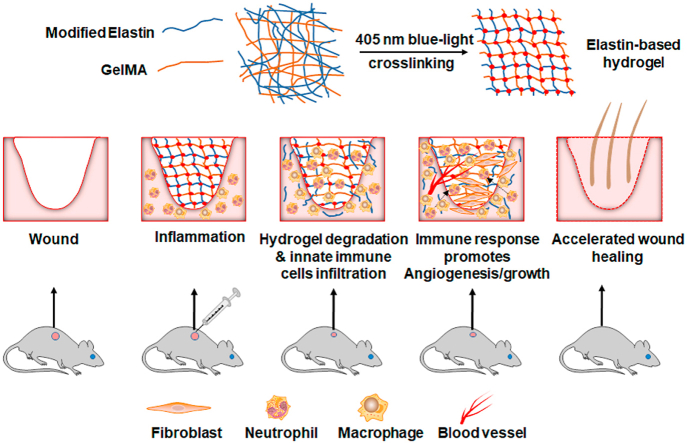

## 1 Introduction

Skin injury is inevitable in human daily life. Especially, the extensive full-thickness injury caused by accidents and fires, is difficult to heal and become a major clinical problem worldwide. At present, approximately 6.5 million people are suffering chronic wounds in the US alone [1]. Wound healing is a complex and orchestrated process which includes sequential and overlapped stages, such as hemostasis, inflammation, angiogenesis, growth, re-epithelialization, and re-modeling [1,2]. Both neutrophils and macrophages are innate immune cells and play vital roles in the stages of inflammation and tissue growth [1,[3], [4], [5], [6]]. As a key player in the innate immune system of the human body, neutrophils are immediately recruited to the wound site after an injury occurs [4]. Neutrophils are not only responsible for the production of reactive oxygen species, antimicrobial peptides and proteases to clear out pathogens and remove cellular debris, but also able to generate cytokines and growth factors to activate angiogenesis and growth stages [6]. On the other hand, macrophages would accumulate at the wound site after neutrophils and normally within 24–48 ​h post-injury [1]. In the early stage of wound healing, macrophages would present pro-inflammatory phenotype (M1) and play active role in the phagocytosis of pathogens. In the late stage of inflammation, the macrophages would be polarized toward an anti-inflammatory phenotype (M2) and promote new vessel formation via expression of pro-angiogenesis growth factors.

Hydrogels have been considered as promising wound dressing materials and regenerative platforms [[7], [8], [9], [10]], as they present highly tunable physicochemical properties and versatility in delivery of bioactive factors or stem cells [[11], [12], [13]]. Additionally, *in-situ* formed hydrogels are in high demand as they can fit uneven wound bed and be used as minimally invasive regenerative system [9,14]. Recently, the development of immunomodulatory biomaterials or hydrogels for wound healing has been attracting great interests [15,16], while the design of hydrogels with intrinsic capacity to modulate innate immune cells (*e.g.* neutrophils or macrophages) for improving wound healing was greatly underappreciated [17].

Elastin is a vital component in the extracellular matrix (ECM) of many organs, such as skin, lung and arteries [2,18]. It is a cross-linked network formed via lysyl oxidase-triggered cross-linking of tropoelastin and confers high elasticity to skin. Fragments of elastin or elastin-derived peptides (EDPs) had shown powerful chemotaxis to various kinds of cells related to wound healing, such as neutrophils, monocytes, macrophages, fibroblasts and endothelial cells [2,[18], [19], [20]]. Furthermore, EDPs can promote the angiogenesis ability of endothelia cells by upregulation of MT1-MMP [19]. Thus, elastin-based biomaterials have been fabricated and applied for wound healing, mainly the elastin precursor-tropoelastin, elastin-like recombinamer (ELR), ECM-derived elastin and acellular dermal matrix [[21], [22], [23], [24], [25], [26], [27], [28]]. For example, Mithieux et al. developed heat-crosslinked tropoelastin implants for point-of-care treatment of open wounds. These off-the-shelf implants could promote new blood vessel formation and accelerate wound repair [22]. Staubli et al. designed multi-sequences containing ELR hydrogels for the loading of adipose tissue-derived stromal cells. These functional hydrogels with engineered channels encouraged vessel and immune cells infiltration in a subcutaneous implant model [23]. As another key component in dermal ECM, collagen and its degraded product gelatin have been combined with elastin to develop biomimetic composites for wound repair [2]. Gelatin presents higher feasibility in modification and process compared to collagen [29]. Currently, only a few studies had been reported on the development of elastin/gelatin-based hydrogels for wound healing [9,30]. Annabi et al. developed visible-light crosslinked sprayable hydrogel adhesive composed of methacrylated tropoelastin, methacrylated gelatin and antimicrobial for wound healing application [9]. Cao et al. engineered composite hydrogels consisting hydrolyzed elastin, gelatin and poly (ethylene glycol) via UV-crosslinking and proved that these hydrogels can guide the behavior of the encapsulated fibroblast [30]. Interestingly, in a recent study, Ibáñez-Fonseca et al. reported that elastin-like recombinamer hydrogels can promote skeletal muscle healing via modulation of macrophage polarization [31]. In the above-mentioned studies, there is still a gap in the deep understanding of the interactions between the elastin-based materials and the innate immune cells during wound healing.

Therefore, in order to elucidate the deep interactions of elastin with innate immune cells in wound healing, *in-situ* formed elastin/gelatin hydrogels were designed and formed via visible-light crosslinking in this work. Due to its good access, elastin derived from bovine ligament was used in current study. Compared to previous pioneer study, current developed hydrogels presented several advances [30]. At first, the elastin was modified with acrylate groups by a fast one-step reaction in mild condition. To minimize the risk of photocrosslinking, the hydrogels were crosslinked via visible-light instead of UV [9,30]. In order to closely mimic the *in vivo* degradation of elastin by elastase (mainly produced by neutrophils) and study the pro-angiogenesis and macrophage modulation effect of the elastase-degraded EDPs, the authors performed *in vitro* co-culture of hydrogel-neutrophils. Then, the derived EDPs-containing conditioned medium was used for *in vitro* modulation of crosslinked M1 macrophage and tube formation study of human umbilical vein endothelial cells (HUVECs). Moreover, the *in vivo* interaction of these hydrogels with neutrophils and macrophages were investigated in mice wound model.

## 2 Materials and methods

All the reagents used in this study were ordered from Beyotime Inc. (Nantong, China) unless otherwise mentioned.

### 2.1 Preparation of soluble elastin

100 ​mg oxalic acid was dissolved in 200 ​mL double distilled H_2_O (ddH_2_O) to prepare 0.5 ​mg/mL oxalic acid solution. Then, 2 ​g insoluble elastin powder derived from bovine neck ligament (Elastin Product Company Inc., Owensville, MO, USA) was added to 40 ​mL 0.5 ​mg/mL oxalic acid solution, then the system was stirred for hydrolysis under 100 ​°C in an oil bath. The supernatant was collected every 2 ​h by centrifugation after cooling. The same volume of oxalic acid solution was added into the remaining undissolved elastin powder for each cycle to continue the hydrolysis. The final soluble elastin was purified by dialysis of the collected supernatant in ddH_2_O for 2 days via using 2 ​kDa MWCO dialysis tubing, then filtering the solution by 0.22 ​μm syringe filter before freeze-drying in a freeze-drier (Pilot2-4M, Biocool Inc, Beijing, China).

### 2.2 Modification of soluble elastin

At first, 0.72 ​g NaH_2_PO_4_ was dissolved in 100 ​mL ddH_2_O to obtain 0.06 ​M NaH_2_PO_4_ buffer solution and 1 ​mol/L NaOH solution was used to adjust the pH to 8.3. Then, 0.1 ​g prepared soluble elastin was dissolved in 10 ​mL the above prepared NaH_2_PO_4_ buffer solution. Following, 40 ​μL Acryloyl- (polyethylene glycol)-NHS (AC-PEG-NHS, MW: 400 da) (Tansh-Tech Inc., Guangzhou, China) was added and the system was reacted for 3 ​h in dark under argon protection. The final Acryloyl-(polyethylene glycol)-Elastin (Elastin-PEG-AC) was obtained after purified by dialysis and then freeze-dried.

### 2.3 Determination of modification rate

Briefly, ninhydrin working solution was prepared by dissolving 100 ​mg ninhydrin and 15 ​mg reduced ninhydrin in 3.2 ​mL dimethyl sulfoxide (DMSO) and adding 1.8 ​mL sodium acetate buffer. The solution was then kept in dark and filled with Argon. The glycine standard solution was prepared at gradient concentration, then 20 ​mg/mL plain elastin solution and modified elastin solution were prepared, respectively. 10 ​μL glycine standard solution or sample (plain elastin or modified elastin) solution was mixed with 200 ​μL ninhydrin reaction solution. The reaction system was then heated in oil bath under 100 ​°C for 20 ​min. After cooling, 1 ​mL 50% ethanol solution was added and the absorbance at 570 ​nm was detected. The modification rate could be calculated according to the following formula:

$$\begin{array}{c} \text{Modification}\;\text{Rate\%}=\\ \frac{\text{Amino}\;\text{group}\;\text{content}\;\text{of}\;\text{elastin}-\text{Amino}\;\text{group}\;\text{content}\;\text{of}\;\text{modified}\;\text{elastin}}{\text{Amino}\;\text{group}\;\text{content}\;\text{of}\;\text{elastin}} \end{array}$$

### 2.4 Fourier-transform infrared spectroscopy (FTIR) characterization

The FTIR spectra were recorded using VERTEX 70v FT-IR Spectrometer (Bruker Instruments Ltd., Billerica, MA, China), ranging from 4000 ​cm^−1^–650 ​cm^−1^. The background correction used the spectrum of air. The elastin and Elastin-PEG-AC were dispersed in ethanol and dripped on the KBr chip. After drying, the KBr chip with sample was put on the sample stage for detection.

### 2.5 Nuclear magnetic resonance spectroscopy (NMR) characterization

The NMR spectra were recorded using 600 ​MHz Ascend NMR Magnets 600 (Bruker Instruments Ltd., Billerica, MA, USA). The AC-PEG-NHS, elastin, and Elastin-PEG-AC were dispersed in deuterium oxide (D_2_O) with 10 ​mg/mL for detection under room temperature.

### 2.6 Preparation of elastin-based hydrogels

At first, 0.5 ​mg/mL lithium phenyl-2,4,6-trimethylbenzoylphosphonate (LAP) solution was prepared by dissolving LAP in 0.01 ​M phosphate buffer saline solution (PBS). Then the above solution was used as a solvent to prepare 100 ​mg/mL methacrylated gelatin (GelMA), plain elastin or modified elastin solution. After mixing the solution according to the different mixing ratios in Table 1, 200 ​μL mixed precursor was placed in a mold (diameter: 5 ​mm) and undergone irradiation under 405 ​nm blue light for 2 ​min to prepare elastin-based and GelMA hydrogels.

**Table 1:** Preparation of elastin/gelatin hydrogels.

Group	Modified elastin solution/μL	GelMA solution/μL	Modified elastin content ratio
GAE-0	0	650	0%
GAE-20	130	520	20%
GAE-50	325	325	50%
GAE-80	520	130	80%
GAE-100	650	0	100%

GAE represents modified elastin (Elastin-PEG-AC) and GelMA composite hydrogels.

### 2.7 Characterization of rheological properties of elastin-based hydrogels

The rheological properties of the hydrogels were tested by a rheometer (MCR 302, Anton Paar Inc., Graz, Austria) at room temperature. 140 ​μL hydrogels precursor solution containing 0.5 ​mg/mL LAP was added to the center of the sample stage, and the upper board was controlled to drop to contact with the precursor solution. The mechanical properties of the precursor solution without blue light irradiation were measured firstly for 50 ​s. Then the blue light irradiation (lamp power 50%) was carried out, and the rheological properties were measured with time.

### 2.8 Characterization of swelling properties of elastin-based hydrogels

The prepared elastin-based hydrogels were swelled in ddH_2_O and weighted. After swelling to constant weight, the hydrogels were freeze-dried, and then further dried at 50 ​°C for 2 ​h to obtain the constant dry weight. The swelling ratio was calculated according to the following Equation, and the swelling curve was drawn.

$$\text{Swelling ​Ratio}=\frac{\text{Wwet}-\text{Wdry}}{\text{Wdry}}$$

Wwet represents the wet weight of the hydrogels and Wdry represents the dry weight of the hydrogels. The equilibrium swelling ratio of hydrogels is the swelling ratio when the hydrogels swell to constant weight.

### 2.9 Enzymatic degradation of elastin-based hydrogels

Elastase from porcine pancreas (Chuangze Biotechnology Inc., Guangzhou, China) and collagenase I from Clostridium histolyticum (Gibco™, Thermo Fisher Scientific, Waltham, MA, USA) were dissolved and diluted to 0.5 ​mg/mL by using PBS. The prepared elastin-based and GelMA hydrogels were immersed in PBS for 12 ​h to reach equilibrium, then each piece of hydrogel was put in 4 ​mL elastase solution after removing the surface liquid by filter paper. Then remaining wet weights of the hydrogels was measured at defined timepoints (0.5, 1, 2, and 4 ​h) after removing surface liquid. Similarly, the enzymatic degradation of the hydrogels in collagenase I solution was performed following protocol mentioned above.

### 2.10 Characterization of ionic strength response of elastin-based hydrogels

The GAE-50 Elastin/Gelatin hydrogels were first swelled in ddH_2_O for 12 ​h then submerged in 0.001 ​M, 0.01 ​M, and 0.1 ​M PBS, respectively, to swell for 12 ​h, and the hydrogels were swelled in ddH_2_O for 12 ​h again, after each step, hydrogels were weighed and images were captured. The changes of volume and weight of the elastin-based hydrogels were analyzed. The variation was represented by the wet weight variation ratio and diameter variation ratio, which can be calculated according to the following equations:

$$\text{Wet ​Weight ​Variation ​Ratio}=\frac{\text{Wm}-\text{Wi}}{\text{Wi}}$$

$$\text{Diameter ​Variation ​Ratio}=\frac{\text{Dm}-\text{Di}}{\text{Di}}$$

Wi represents the original weight of hydrogels and Wm represents the weight of hydrogels after swelling. Di and Dm represent diameters of the original hydrogels and swelled hydrogels, respectively.

### 2.11 Scanning electron microscope (SEM) imaging

The microstructure of the elastin-based and GelMA hydrogels was characterized by a SEM (Phenom Pharos G1, Netherlands). The prepared hydrogels were frozen by liquid nitrogen at first prior to lyophilization for two days. The freeze-dried hydrogels were coated with gold before observation in the SEM.

### 2.12 Cell culture

Primary human umbilical vein endothelial cells (HUVECs) were obtained from American Type Culture Collection (ATCC, Manassas, VA, USA), and cultured in ECM medium containing 10% fetal bovine serum, 1% endothelial cell growth supplement (ECGs) with 1% penicillin-streptomycin solution (ScienCell Research Laboratories Inc., Carlsbad, CA, USA). The human foreskin fibroblast (HFF-1) was provided by Stem Cell Bank (Chinese Academy of Sciences) and maintained in DMEM growth medium (Gibco™, Thermo Fisher Scientific, Waltham, MA, USA) supplemented with 15% fetal bovine serum (FBS), 1% penicillin-streptomycin solution. Raw 264.7 was obtained from Stem Cell Bank (Chinese Academy of Sciences, Shanghai, China) and cultured in RPMI 1640 (Gibco™, Thermo Fisher Scientific, Waltham, MA, USA) medium with 10% fetal bovine serum (FBS), 1% penicillin-streptomycin solution. All cells were tested for bacteria, fungi, mycoplasma and virus contamination before experiments, and were kept at 37 ​°C in a humidified atmosphere containing 5% CO_2_.

### 2.13 Cytotoxicity of the precursors of elastin-based hydrogels

Elastin-PEG-AC and GelMA were dissolved in 0.01 ​M PBS to prepare 10 ​wt% solutions, respectively. Then they were filtered by 0.22 ​μm filter membranes. Elastin-PEG-AC and GelMA solutions were mixed in 1:1 ratio to form 100 ​mg/mL GAE-50 precursor solutions. Next, they were diluted into 40, 20, 10 and 1 ​mg/mL working solutions for cell culture. For the dilution, sterilized PBS and complete medium (Gibco Inc., USA) were used and the final volume ratio of the PBS and complete medium was fixed to 1:1 for each formulation. 1 ​mL PBS and 1 ​mL complete medium were mixed to for the culture of the control group. The precursor working solutions were applied for the culture of HFF-1 ​cells. At first, HFF-1 ​cells were cultivated for 24 ​h for cell adhesion. Then, original medium was discarded and 200 ​μL precursors solution or PBS/medium solution was added into each well. After co-cultivating for 24 ​h or 48 ​h, cell activity was detected by using the CCK-8 solution and the absorbance at 405 ​nm was recorded. The cell viability was calculated according to the following formula:

$$\text{Cell}\;\text{viability\%}=\left({\text{OD}}_{\text{E}}{-\text{OD}}_{\text{B}}\right)\text{/}\left({\text{OD}}_{\text{PBS}}{-\text{OD}}_{\text{B}}\right)\text{×}100\text{\%}$$

OD_E_ represents the absorbance of the experimental group, OD_B_ represents the absorbance of the blank group, which added CCK-8 without cells, OD_PBS_ represents the absorbance of the control group.

### 2.14 Cell adhesion assay

At first, the precursor solutions of the GAE-0, GAE-20 and GAE-50 groups were added into the wells of the 96-well plate (100 μL/well), respectively. Following, the solutions were undergone blue light irradiation for 5 ​min to form the hydrogels. Then, the formed hydrogels were hydrated in 100 ​μL complete medium for 1 ​h. After removing the medium in the well, 100 ​μL HFF-1 ​cell suspension (800,000/mL) was added onto the surface of the hydrogel in each well. Afterwards, the cells were culture at 37 ​°C, and then CCK-8 assay were conducted at 24, 48 and 72 ​h, respectively.

### 2.15 Isolation of peripheral blood neutrophils and EDP-conditioned medium

Since there is very small amount of peripheral blood can be obtained from the mouse without sacrifice each time (<180 μL/mouse), it is hard to get enough neutrophils from mouse blood for this study. Additionally, a recent study reported that elastase from human neutrophils was able to selectively kill cancer cells from mouse [32]. Therefore, human blood from donor was employed in current study for isolation of neutrophiles and subsequent use in co-culture with elastin-based hydrogels, in order to get the EDPs-containing conditioned medium (EDP-conditioned medium). Briefly, fresh whole blood treated with an anticoagulant was obtained from donor with informed consent and stored in a 15 ​mL centrifuge tube. Then, the polymorphprep™ (Axis-shield, Alere Technologies AS, Oslo, Norway) working solution with same volume as the blood was carefully added into the blood. All procedures involved in the human participants were reviewed and approved by the Ethics Committee of the seventh affiliated hospital of Sun Yat-sen university. Centrifuge the tubes at 800 ​g for 30 ​min in a swing-out rotor at 18–22 ​°C. After centrifugation, the top band at the sample interface will consist of mononuclear cells and the lower band of polymorphonuclear cells (PMNs). The PMNs were collected in a clean centrifuge tube and suspended in red blood cell lysis buffer for 2 ​min under room temperature, finally cultured in RPMI 1640 complete medium. The hydrogels, including GAE-0, GAE-20 and GAE-50 groups, were placed into the culture of neutrophils for 24 ​h to allow the elastase to degrade the hydrogels. By using this model, the elastin derived peptides (EDPs) were released into the medium (termed EDP-conditioned medium) which was collected and use in the following studies.

### 2.16 Macrophage polarization and modulation by EDP-conditioned medium

For M1 polarization, LPS (1000 ​ng/mL) (Sigma-Aldrich, St. Louis, MO, USA) was supplemented to the culture of Raw 264.7 ​cells for 24 ​h. For M2 polarization, Raw 264.7 ​cells were stimulated by IL-4 (20 ​ng/mL), IL-13 (20 ​ng/mL) (Peprotech, Rocky Hill, NJ,USA) for 24h. For the modulation study by EDP-conditioned medium, 2 ​mL EDP-conditioned mediums were added into each well of the 24-well plate for the culture of M1 macrophages.

### 2.17 Capillary tube formation under EDP-conditioned medium

96-well plate and 200 ​μL tips were placed in a refrigerator for 30 ​min, and Matrigel (Corning, New York, USA) were unfrozen at 4 ​°C on ice prior to pipetting 60 ​μL into each well. Then the 96-well plate was incubated at 37 ​°C and 5% CO2 for 60 ​min to solidify Matrigel. The HUVECs density was adjusted to 100,000 ​cells/mL and resuspended in 100 ​μL various EDP-conditioned mediums to observe tube formation. Images were captured using an inverted light microscope (Zeiss inverted microscope Primovert) at 40x magnification after 6 ​h and 10 ​h, respectively. Images from 3 samples per group were used for quantitative tube formation analysis via ImageJ software.

### 2.18 RNA isolation and quantitative real-time polymerase chain reaction (PCR)

Total RNA was isolated from macrophages by using TRIzol reagents (Invitrogen, Carlsbad, CA, USA), and the reverse transcription of first-strand cDNA was performed by using a PrimeScript®RT reagent kit (Takara, Kyoto, Japan) according to the manufacturer's protocol. Quantitative real-time-PCR analyses for mRNA of TGF-β, IL-4, IL-10, iNOS, TNF-α, IL-6 and β-actin were performed by using SYBR Green PCT Master Mix (Takara, Kyoto, Japan). The mRNA level of β-actin was used as an internal control. Table 2 listed the oligonucleotide sequences for PCR assay.

**Table 2:** The sequences of oligonucleotides used for PCR.

Gene	Forward Primer	Reverse Primer
TGF-β	GAGCCCGAAGCGGACTACTA	TGGTTTTCTCATAGATGGCGTTG
IL-4	CCCCAGCTAGTTGTCATCCTG	CAAGTGATTTTTGTCGCATCCG
IL-10	GCTGGACAACATACTGCTAACC	ATTTCCGATAAGGCTTGGCAA
iNOS	CAGCTGGGCTGTACAAACCTT	CATTGGAAGTGAAGCGTTTCG
TNF-α	GCTCTTCTGTCTACTGAACTTCGG	ATGATCTGAGTGTGAGGGTCTGG
IL-6	CTCCGACTTGTGAAGTGGTAT	CACCTCAATGGACAGAATATCA
β-actin	CCACCATGTACCCAGGCATT	AGGGTGTAAAACGCAGCTCA

### 2.19 Construction of full-thickness skin defect model in mouse

To evaluate the wound repair potential of the elastin-based hydrogels, full-thickness skin round wound model was created on the back of female C57BL/6J mice in this study. All animals were kept and fed in a pathogen-free environment. Procedures for the care and use of animals were approved by the China Technology Industry Holdings (Shenzhen, China) Co. Ltd. and followed all applicable institutional and government regulations regarding the ethical use of animals. Female C57BL/6J mice weighing about 20 ​g and 8 weeks age were used for studies. All mice were divided randomly into 4 groups: control, 0% elastin hydrogel (GAE-0), 50% elastin hydrogel (GAE-50) and 100% elastin hydrogel (GAE-100). Each group contained 4 mice for each timepoint (Day 3, 7 and 14) and all mice were acclimatized for 1 week before the experiments. Before surgery, all mice were anaesthetized with isoflurane and shaved on the back with an electric clipper, then the wound site was sterilized with 70% ethanol. Full-thickness skin wounds of 10 ​mm diameter were generated using a biopsy punch, then hydrogel precursor solutions of different formulations (total concentration 100 ​mg/mL) were injected to the wound site. Following, 405 ​nm blue-light cross-linking was conducted for 2 ​min to prepare *in-situ* formed hydrogels in the wound defect. Afterwards, all the wounds were covered with breathable patches, followed by bandaging with gauze pad. The mice were sacrificed on day 7 and 14 after surgery and skin tissues at the wound site were retrieved and fixed in 10% formalin for 24 ​h for histological and immunohistochemistry analysis.

### 2.20 Analysis of wound closure rate

The images of wounds were obtained on day 0, 3, 7, 14 post-surgery. The areas of wounds were analyzed by ImageJ. The wound closure rate was calculated to show the size change on each distinct stage using the formula below:

$$\text{Wound}\;\text{healing}\;\text{rate}=\left({\text{S}}_{\text{0}}{-\text{S}}_{\text{A}}\right)\text{/S0×}100\text{\%}$$

S_0_ represents the wound area of day 0, S_A_ represents the wound area of image-capturing day.

### 2.21 Histological staining and histomorphological analysis

Full-thickness skin samples of mice were collected along the outer edge of the entire wound on 3rd, 7th and 14th day. Tissues were fixed with 4% paraformaldehyde for 24 ​h, and then embedded in paraffin to be sectioned to 45 ​μm thickness slices, finally slices were stained with Hematoxylin-Eosin (HE) and Masson's trichrome. Collagen deposition in wound area was quantified by using the Masson's trichrome staining images. All slices were photo-captured by microscope (IX53, Olympus, Japan). Slides from 3 samples per group were used for quantitative collagen deposition analysis via ImageJ software.

### 2.22 Immunohistochemistry staining

The paraffin section was cut into 5 ​μm thin layer. Each section was incubated with 10% goat serum for 1 ​h at 37 ​°C, followed by incubation with primary antibodies (ABclonal, Wuhan, China) overnight at 4 ​°C and secondary antibodies (Invitrogen, Manassas, Virginia, USA) for 2 ​h at 37 ​°C. Neutrophil marker F4/80, endothelial cell marker CD31 and macrophages (including M1 and M2) markers CD86, CD206 were chosen in our experiment. Images were captured by microscope (IX53, Olympus, Japan) and images from 3 samples per group were used for quantitative analysis via ImageJ software.

### 2.23 Immunofluorescence staining

To assess the effects of the elastin-based hydrogels on angiogenesis and macrophages modulation, immunofluorescence staining of CD31, F4/80, CD86, CD206 on wound tissues were performed day 3, 7 and 14 after surgery. Freezed slices were fixed with 4% paraformaldehyde for 10 ​min, then washed with PBS for 3 times, followed by blocked with goat serum containing 0.1% Triton. Afterwards, the slides were incubated overnight with anti-CD31, F4/80, CD86, CD206 antibodies (1:100, Abconal, China) at 4 ​°C, followed by incubation with goat anti-mouse Alexa Fluor® 647-conjugated secondary antibody (Abcam, Cambridge,England) for 1 ​h at room temperature. Finally, slices were stained with DAPI for 5 ​min and washed with PBS for 3 times. The images were acquired by using a fluorescence microscope (Leica DM6 B, Wetzlar, Germany) and images from 3 samples per group were used for quantitative analysis via ImageJ software.

### 2.24 Statistical analyses

The data in this research were analyzed using the One-tailed unpaired *t*-test or One-way Analysis of Variance followed by Dunnett's post hoc test. *P* ​< ​0.05 was considered to be statistically significant. All the experiments were operated at least 3 times with more than 3 samples for each test.

## 3 Results

### 3.1 Chemical structure and preparation of elastin-based hydrogels

**Fig. 1:**
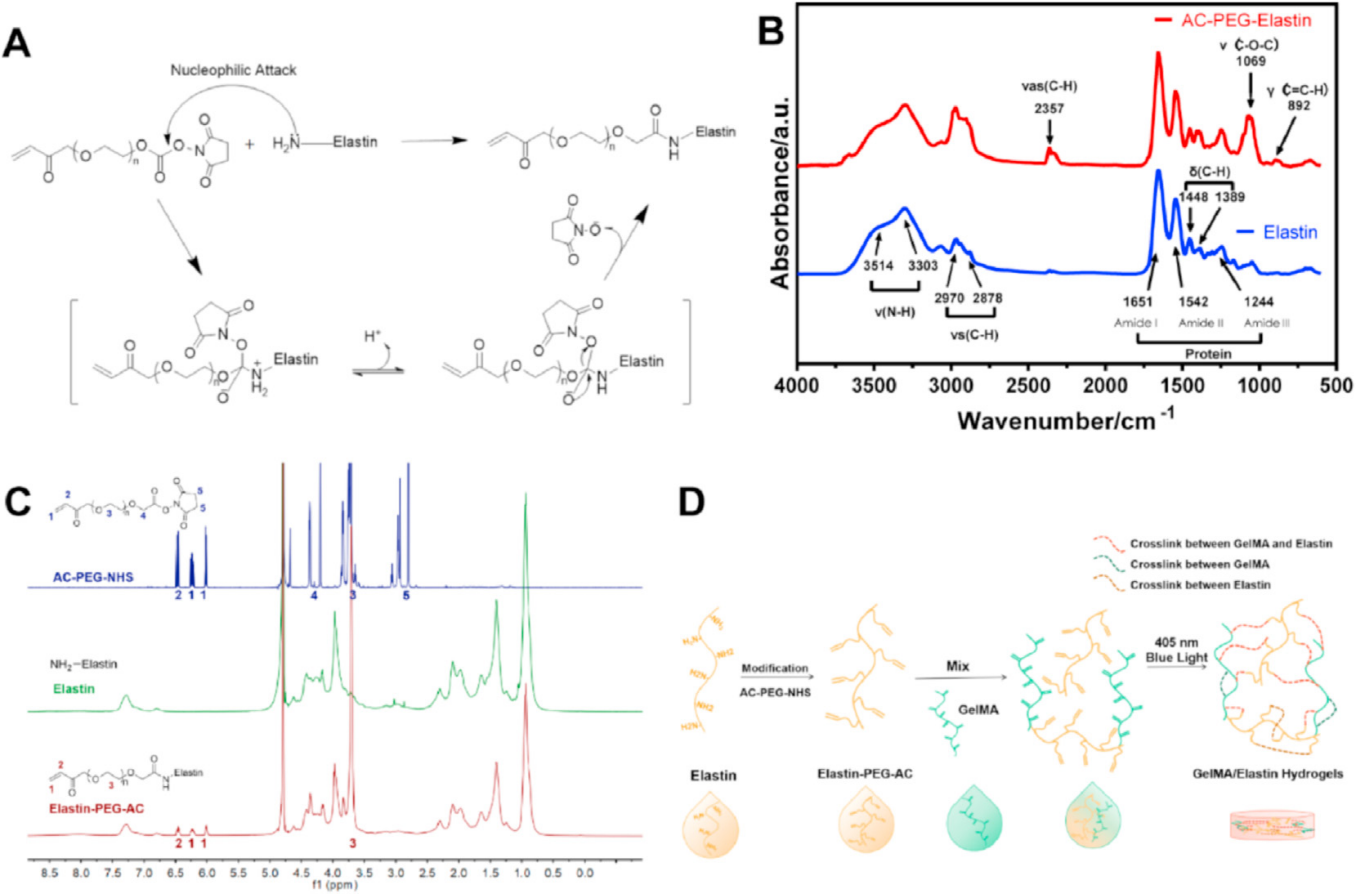
**Chemical characterizations of modified elastin and preparation scheme of the elastin-based hydrogels. (A)** The modification mechanism of the primary amino groups on elastin. **(B)** FTIR spectra of elastin and Elastin-PEG-AC. Here, v represents stretching vibration, vs represents symmetric stretching vibration, vas represents unsymmetric stretching vibration, δ represents in-plane deformation vibration and γ represents out-plane deformation vibration. (C) ^1^H NMR (600 ​MHz; D_2_O) spectra of AC-PEG-NHS, elastin and Elastin-PEG-AC. **(D)** Preparation scheme of the elastin-based hydrogels.

AC-PEG-NHS can modify elastin via nucleophilic attack and graft the carbon-carbon double bonds to the elastin as showed in Fig. 1A. A modification rate of the amino groups in elastin up to 93.91 ​± ​1.54% was obtained via ninhydrin test. To further confirm the grafting, the chemical structures of the plain elastin and Elastin-PEG-AC were characterized by FITR (Fig. 1B). Amide absorbing region Ⅰ, Ⅱ, and Ⅲ at 1651 ​cm^−1^, 1542 ​cm^−1^ and 1244 ​cm^−1^ respectively can be detected in both proteins [33]. The peaks located at 3514 ​cm^−1^ and 3330 ​cm^−1^ are related to the stretching vibration of the N–H bond [34], which had no obvious changes after modification. This is because the N–H structure is still retained after modification and is abundant in the protein skeleton. However, the increased signal of symmetric stretching vibration of C–H at 2970 ​cm^−1^ and 2878 ​cm^−1^ and the appearance of C–H unsymmetrical stretching vibration at 2357 ​cm^−1^ [35], C–O–C stretching band at 1069 ​cm^−1^ [36], and the out-plane deformation vibration of C–H at 892 ​cm^−1^ indicate the successfully grafting of the double carbon bonds with PEG chain to the elastin.

Additionally, the ^1^H NMR analysis was performed to characterize the chemical structure of AC-PEG-NHS, elastin and Elastin-PEG-AC (Fig. 1C). The 6 characteristic peaks of AC-PEG-NHS appeared at 6.0 ​ppm (-CH=C**H**_2_), 6.2 ​ppm (-CH=C**H**_2_), 6.5 ​ppm (-C**H**=CH_2_-), 3.7 ​ppm (–OCH_2_CH_2_–), 4.4 ​ppm (–OCH_2_COO–), and 2.8 (–CH_2_CH_2_– in N-succinimidyl) respectively. After modification, the Elastin-PEG-AC showed 4 new peaks corresponding to (-CH=CH_2_) and (–OCH_2_CH_2_–), and similar peaks of non-modified elastin, but without the peaks of (–OCH_2_COO–) and (–CH_2_CH_2_– in N-succinimidyl). These results clearly confirmed the successful conjugation of acrylate groups onto elastin and the Elastin-PEG-AC was purified very well without AC-PEG-NHS residual.

### 3.2 Elastin-based hydrogels presented modulable physicochemical characteristics

**Fig. 2:**
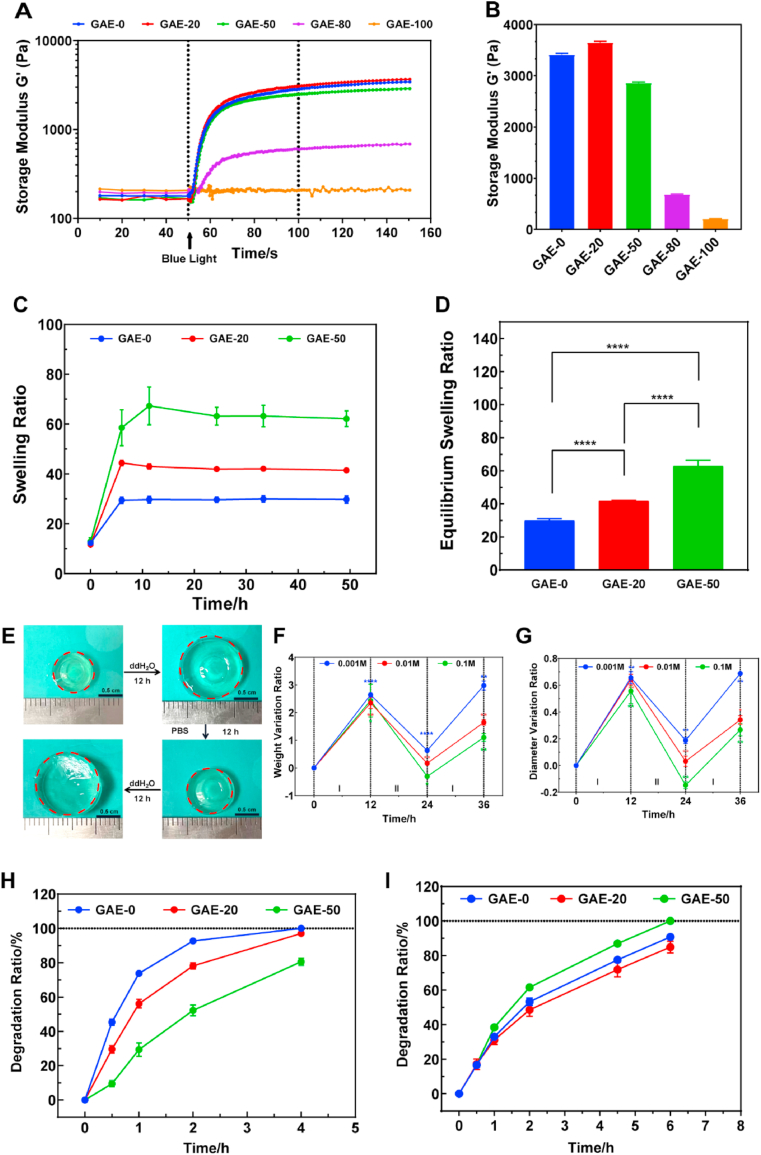
**Physicochemical characterizations of elastin-based hydrogels. (A)** Storage modulus G′ profiles of the elastin-based hydrogels recorded from precursor solutions to hydrogels formation. **(B)** Storage modulus G′ of elastin-based hydrogels with different elastin ratio. **(C)** Swelling profiles of the elastin-based hydrogels within 48 ​h. **(D)** Equilibrium swelling ratio of the elastin-based Hydrogels. **(E)** Images of elastin-based hydrogels responding to the variation of the ion concentration. **(F)** The weight variation of the hydrogels during alternative immersion of the hydrogels in ddH_2_O (I) and PBS (II). **(G)** The diameter variation of the hydrogels during alternative immersion of the hydrogels in ddH_2_O (I) and PBS (II). PBS of 0.1 ​M, 0.01 ​M and 0.001 ​M concentrations were tested in **(F)** and **(G)**. **(H)** Enzymatic degradation profile of the elastin-based hydrogels in 0.5 ​mg/mL elastase PBS solution at 37 ​°C. **(I)** Enzymatic degradation profile of the elastin-based hydrogels in 0.5 ​mg/mL collagenase PBS solution at 37 ​°C. GAE-0: Methacrylated gelatin (GelMA). GAE-20, GAE-50, GAE-80, GAE-100: GelMA, composite hydrogels containing 20%, 50%, 80% and 100% modified elastin (wt.%), respectively. N ​= ​3. ∗*P* ​≤ ​0.05, ∗∗*P* ​≤ ​0.01, ∗∗∗∗P ​≤ ​0.0001. In (**F, G**), each variation ratio was compared to the last tested ratio.

The rheological properties of elastin-based hydrogels with different elastin ratios were assessed by a rheometer (Fig. 2A and B). The storage moduli of the hydrogels increased remarkably after the onset of the blue-light exposure, which represents the beginning of the hydrogel cross-linking. The storage modulus of the GAE-0, GAE-20 and GAE-50 hydrogels presented comparable storage moduli ranged from around 2700 to 3400 ​Pa. However, when the elastin ratio increased to 80% and 100%, the storage modulus dropped obviously to 645 ​Pa and 208 ​Pa, respectively. The inferior mechanical properties of GAE-80 and GAE-100 may bring difficulties in the following evaluations, such as swelling and cell culture. Hence, this study would focus on the mechanically comparable GAE-0, GAE-20 and GAE-50 groups for the further *in vitro* experiments, and then select the best group based on *in vitro* evaluation for *in vivo* studies.

The swelling results showed that all the elastin-based hydrogels reached their swelling equilibrium within 24 ​h (Fig. 2C). The equilibrium swelling ratio increased when increasing elastin ratio (Fig. 2D). In a further study, it was found that the elastin-based hydrogels showed ion-concentration response, namely they swelled dramatically in ddH_2_O and shrank in 0.01 ​M PBS (Fig. 2E). While recording the weight and diameter of the hydrogels, it was found that the diameter and weight of the elastin-based hydrogels can change alternatively responding to the alternative immersion in ddH_2_O and PBS of varied concentrations (Fig. 2F and G). After the first immersion in PBS and the second immersion in ddH_2_O, both the weight and diameter variation ratios showed inverse relation to the ion concentrations of PBS, with the hydrogels in 0.001 ​M PBS presented the highest variation ratios (Fig. 2F and G).

The elastin-based hydrogels degraded in the presence of elastase (Fig. 2H). Surprisingly, the GAE-0 presented highest degradation ratio among the groups. Both GAE-0 and GAE-20 degraded completely after 4 ​h. While GAE-50 showed remarkable stability in elastase degradation to the rest groups and only reach around 80% degradation after 4 ​h. Furthermore, the stability of the hydrogels was tested in collagenase I solution. In collagenase I solution, the hydrogels showed similar degradation profiles, with GAE-50 presented slightly higher degradation ratio compared to the rest groups. GAE-50 degraded completely after 6 ​h, while the rest groups reached above 80% degradation ratio after 6 ​h (Fig. 2I).

**Fig. 3:**
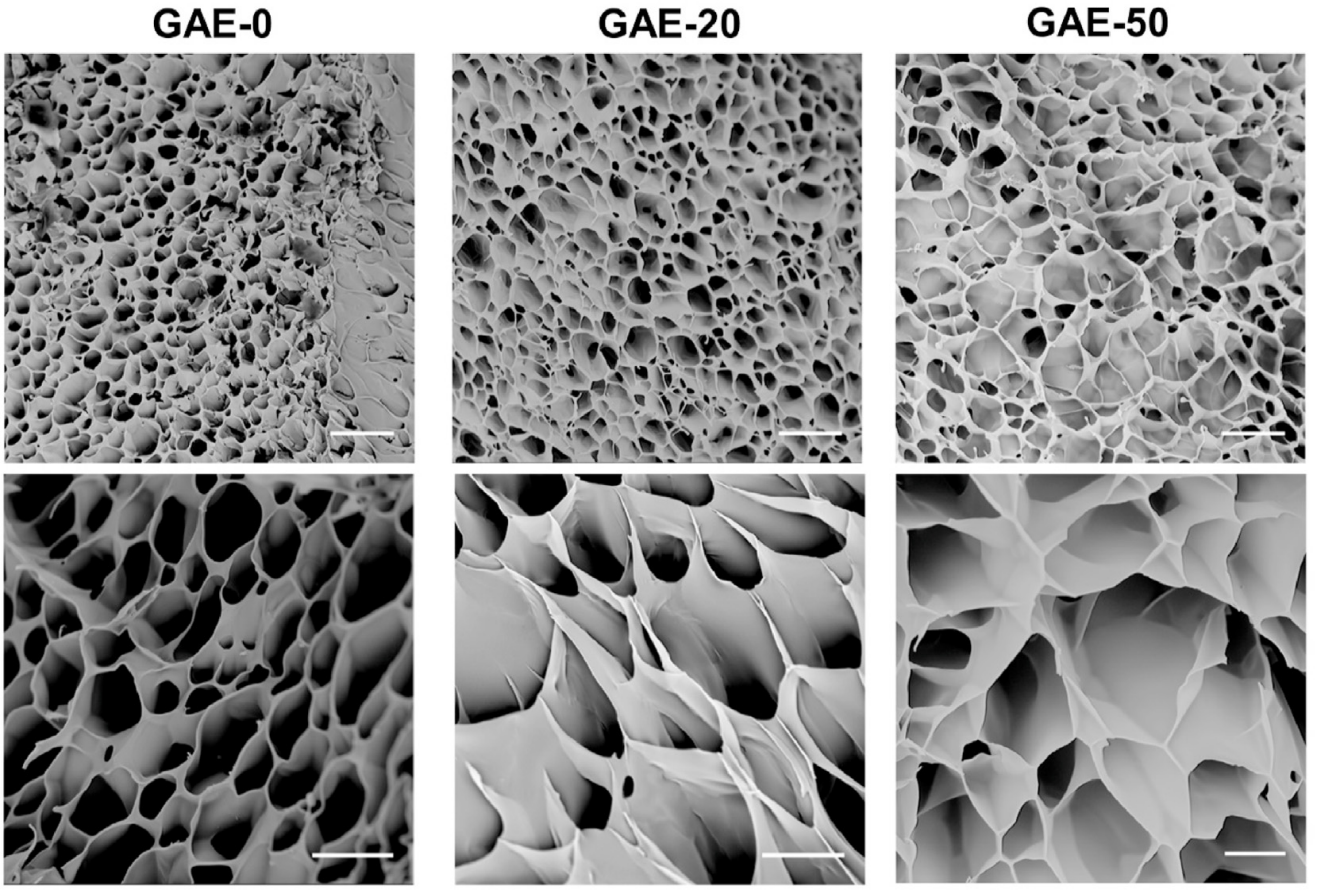
**Morphology characterization of the elastin-based hydrogels by SEM**. GAE-0: GelMA. GAE-20, GAE-50: GelMA, composite hydrogels containing 20% and 50% modified elastin, respectively. Scale bar:50 ​μm (upper), 20 ​μm (lower).

The morphology of elastin-based hydrogels was observed by SEM. From Fig. 3, it was found that all the hydrogels possessed honeycomb-like porous and interconnected structure. The pore sizes of the various hydrogels were similar and ranged from around 20 to 60 ​μm. The GAE-100 also presented porous structure but with pore size less than 20 ​μm (Supplementary Figure 1).

### 3.3 Cytotoxicity of the elastin-based hydrogel and precursors

After culture on the surface of the hydrogels for 24 ​h, it was found that the HFF-1 can adhere to the surface of the hydrogels. However, only GAE-50 group supported the spreading of HFF-1, and the rest groups showed cell aggregation (Supplementary Figure 2A). Regarding the viability, though no obvious cell proliferation was recorded, the cells maintained similar viability over 72 ​h and no significant differences among the hydrogel groups (Supplementary Figure 2B). The cytotoxicity of the precursors of GAE-50 was further evaluated and the results showed that the cell viability percentages in all the tested concentration were over 100% after 48 ​h co-culture (Supplementary Figure 2C). Moreover, the cell viability can reach over 200% after 48 ​h cultivation with 20 ​mg/mL precursor. These results indicated that the precursors of the elastin-based hydrogels were non-cytotoxic. The cell attachment behavior and subsequent cell viability may relate to the surface roughness or topological structure of the hydrogels and will be investigated deeply in the future.

### 3.4 EDP-conditioned medium promoted M1 and M2 polarization of macrophages *in vitro*

**Fig. 4:**
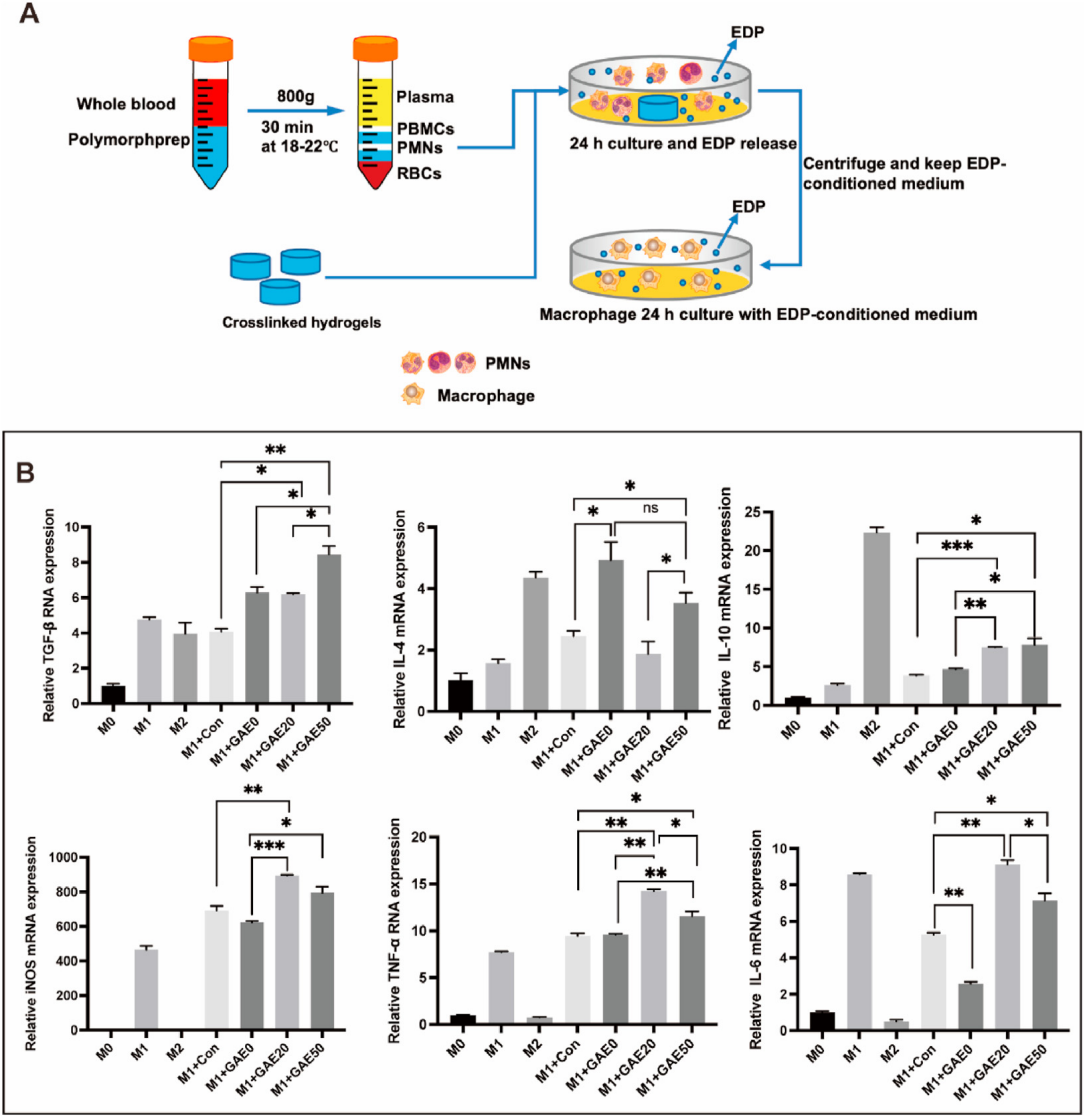
**The *in vitro* macrophage modulation effect of the EDP-conditioned medium. (A)** Preparation scheme of the EDP-conditioned medium by co-culture of neutrophils and the elastin-based hydrogels. **(B)** Relative mRNA expression of markers of M2 (TGF-β, IL-4, IL-10) and M1 (iNOS, TNF-α, IL-6) macrophages cultured in EDP-conditioned medium for 24 ​h. M0: Raw 264.7 macrophages of M0 phenotype. M1: Raw 264.7 macrophages of M1 phenotype. M2: Raw 264.7 macrophages of M2 phenotype. M1+con: M1 macrophages cultured in conditioned medium from pure neutrophils culture. M1+GAE0, M1+GAE20, M1+GAE50: M1 macrophages cultured in conditioned medium from co-culture of neutrophils with GelMA, composite hydrogels containing 20% and 50% modified elastin, respectively. N ​= ​3. ∗*P* ​≤ ​0.05, ∗∗*P* ​≤ ​0.01, ∗∗∗*P* ​≤ ​0.001.

In order to mimic the *in vivo* generation of EDPs by elastase degradation and the modulation effect of EDPs on macrophages, the elastin-based hydrogels were co-cultured with neutrophils *in vitro* to produce EDPs and EDP-conditioned medium (Fig. 4A). Then, the EDP-conditioned medium was used for the culture of M1 macrophages and the mRNA expression levels of M2 associated markers (TGF-β, IL-4, IL-10) and M1 associated marker (iNOS, TNF-α, IL-6) were analyzed (Fig. 4A and B). Fig. 4B showed that the EDP-conditioned mediums increased the expressions of both M1 and M2 macrophages markers. In terms of the expression of M2 macrophage markers, conditioned mediums of all the hydrogel groups triggered significant expression of TGF-β compared to the control, but the one of GAE-50 outdid those of the other two groups. The conditioned mediums of GAE-0 and GAE-50 outperformed the control in IL-4 expression, but no significant difference between the two groups. Both the conditioned mediums of GAE-20 and GAE-50 induced remarkable IL-10 expression compared to control and the one of GAE-0. Regarding the expression of M1 macrophage markers, both the conditioned mediums of GAE-20 and GAE-50 led to higher expression of iNOS, TNF-α, and IL-6 compared to the control and the one of GAE-0. But the conditioned medium of GAE-50 induced inferior expression of TNF-α, and IL-6 compared to GAE-20. Therefore, the EDP-conditioned medium of GAE-50 increased the expression of M2 associated markers and decreased the expression of M1 associated compared to the one of GAE-20.

### 3.5 EDP-conditioned medium displayed proangiogenic effect *in vitro*

**Fig. 5:**
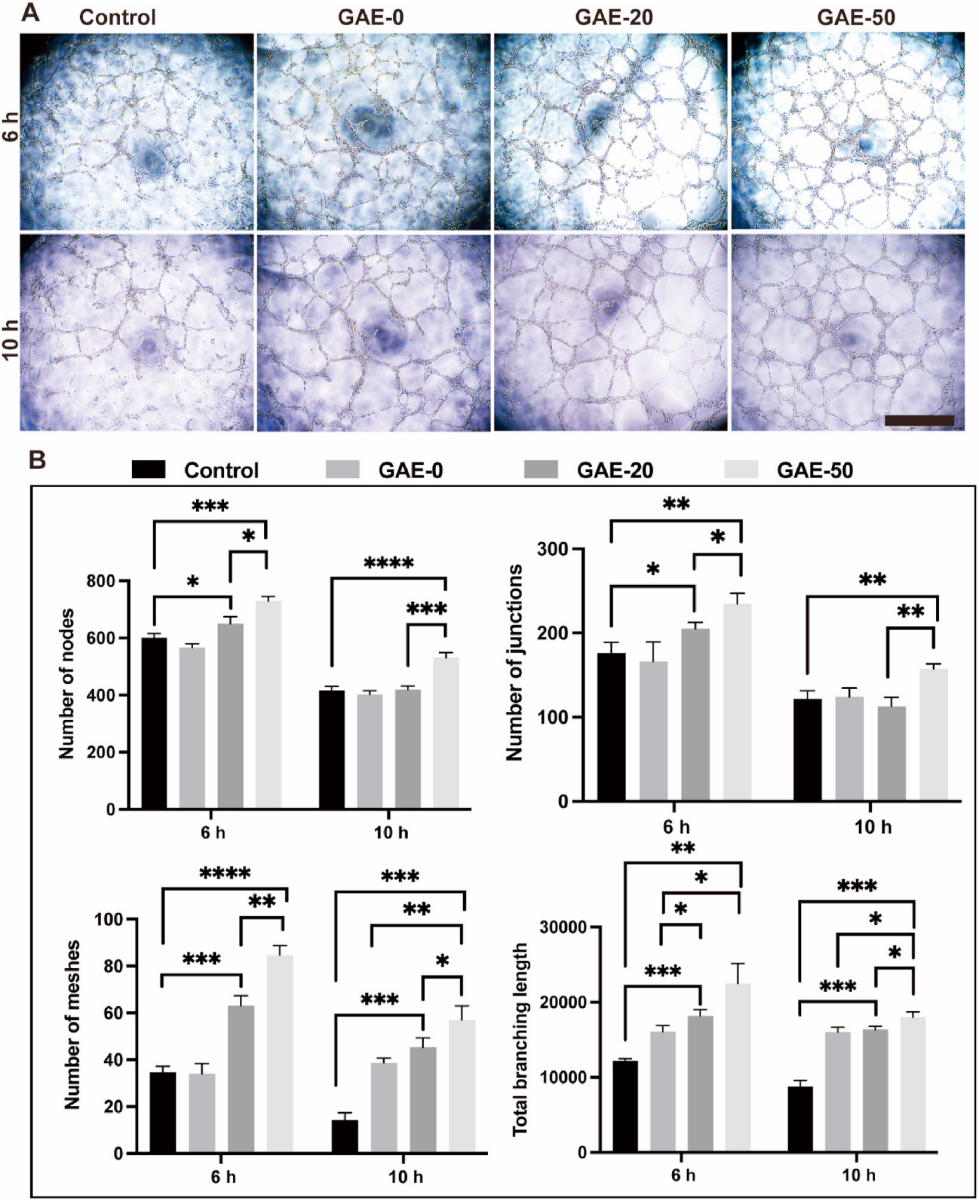
**The *in vitro* proangiogenic effect of the EDP-conditioned medium on HUVECs cultured in Matrigel. (A)** Tube formation images of HUVECs cultured in Matrigel for 6 and 10 ​h, supplemented with EDP-conditioned medium obtained by co-culture of neutrophils and the elastin-based hydrogel. Control: HUVECs cultured in conditioned medium from pure neutrophils. GAE-0, GAE-20 and GAE-50: HUVECs cultured in conditioned mediums from co-culture of neutrophils with GelMA hydrogels, composite hydrogels containing 20% and 50% modified elastin, respectively. Scale bar: 400 ​μm. **(B)** Quantitative analysis of the formed nodes, junctions, meshes and branching length. N ​= ​3. ∗*P* ​≤ ​0.05, ∗∗*P* ​≤ ​0.01, ∗∗∗*P* ​≤ ​0.001, ∗∗∗∗*P* ​≤ ​0.0001.

The EDP-conditioned mediums were further employed in the culture of HUVECs in Matrigel model to assess the proangiogenic effects of the elastin-based hydrogel (Fig. 5). Tube formation of HUVECs supplemented with EDP-conditioned medium was observed after treatment for 6 and 10 ​h (Fig. 5A). Fig. 5A showed that all the groups showed tube formation at both timepoints. All the groups showed the maximum number of the tubes after 6 ​h, then the number of the tubes gradually decreased as time due to the merging of the tubes. It is obviously to find that the conditioned mediums of the elastin-based hydrogels induced better tube formation performance compared to the control. Fig. 5B presented the quantitative analysis of the tube formation of the groups. The conditioned mediums of the elastin-based hydrogels induced larger numbers of nodes, junctions, meshes and total branching length after 6 ​h, and larger number of meshes after 10 ​h, compared to the control and the one of the GAE-0. While the conditioned medium of the GAE-50 group exceeded the rest groups in all these parameters related to tube formation at both timepoints.

### 3.6 Elastin-based hydrogels improved wound contraction *in vivo*

**Fig. 6:**
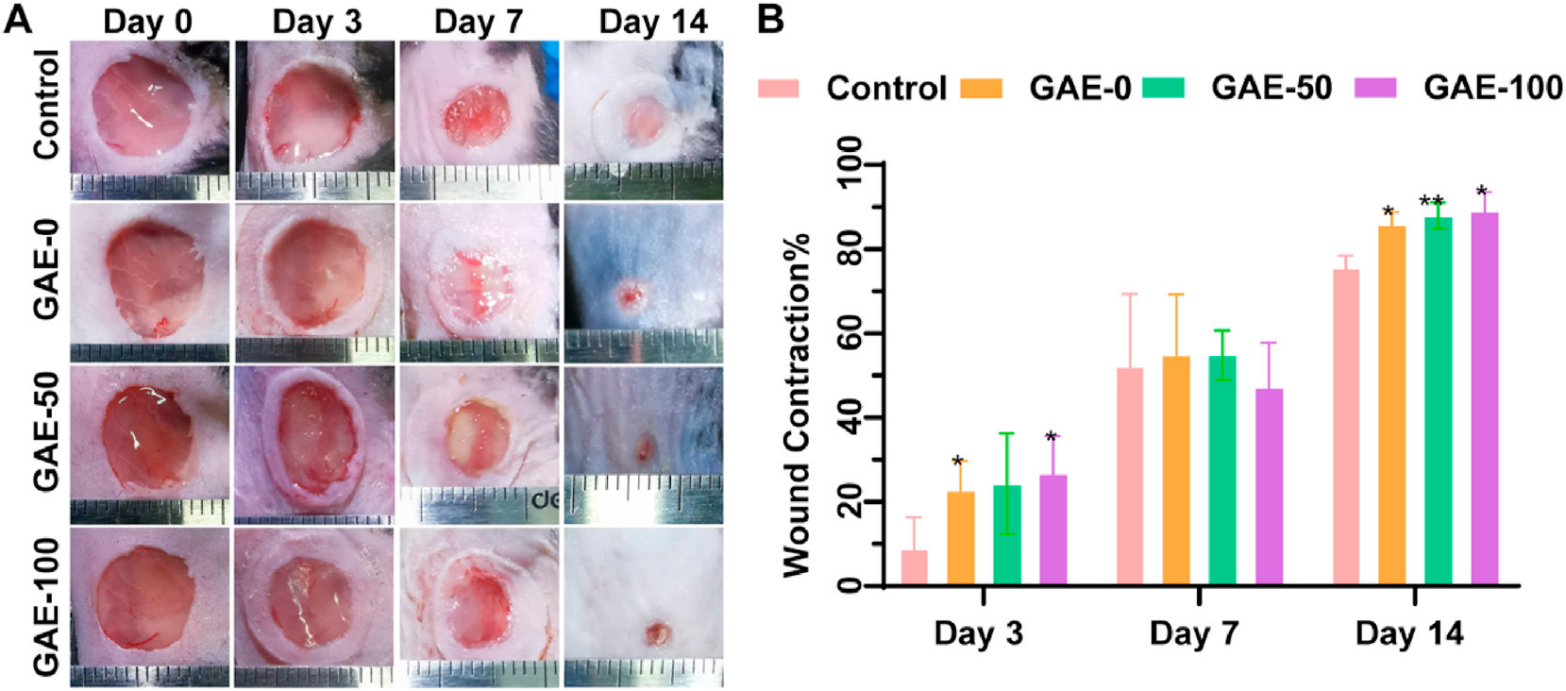
**The influence of elastin-based hydrogels treatment on wound contraction in *vivo* mice wound model. (A)** Images of wound healing procedure treated by the elastin-based hydrogels for 3, 7 and 14 days. **(B)** Wound contraction percentage analysis by quantification of the decreased wound area at different timepoints. Control: Wounds without hydrogel treatment. GAE-0, GAE-50, GAE-100: Wounds treated by GelMA hydrogels, composite hydrogels containing 50% and 100% modified elastin, respectively. N ​= ​3. ∗*P* ​< ​0.05, ∗∗*P* ​< ​0.01, compared to control group at the same timepoints.

The wound regeneration potential of the elastin-based hydrogels was evaluated in a full-thickness mice skin wound model. Fig. 6A showed the representative images of wound area after the treatment of the GAE-0, GAE-50, GAE-100 and control. Obvious inflammation response was not observed in all the groups, and wound areas of all the groups gradually decreased with time. After 14 days, the hydrogels treated groups showed nearly completely wound healing in the epidermal, while the control still present obvious wound area. Fig. 6B showed the quantitative analysis of the wound contraction ratio. The GAE-0 and GAE-100 groups was superior to the control on day 3. Though there were no significant differences among all the groups on day 7, all the hydrogel groups presented better wound contraction compared to the control on day 14.

### 3.7 Elastin-based hydrogels promoted dermal regeneration and collagen deposition

**Fig. 7:**
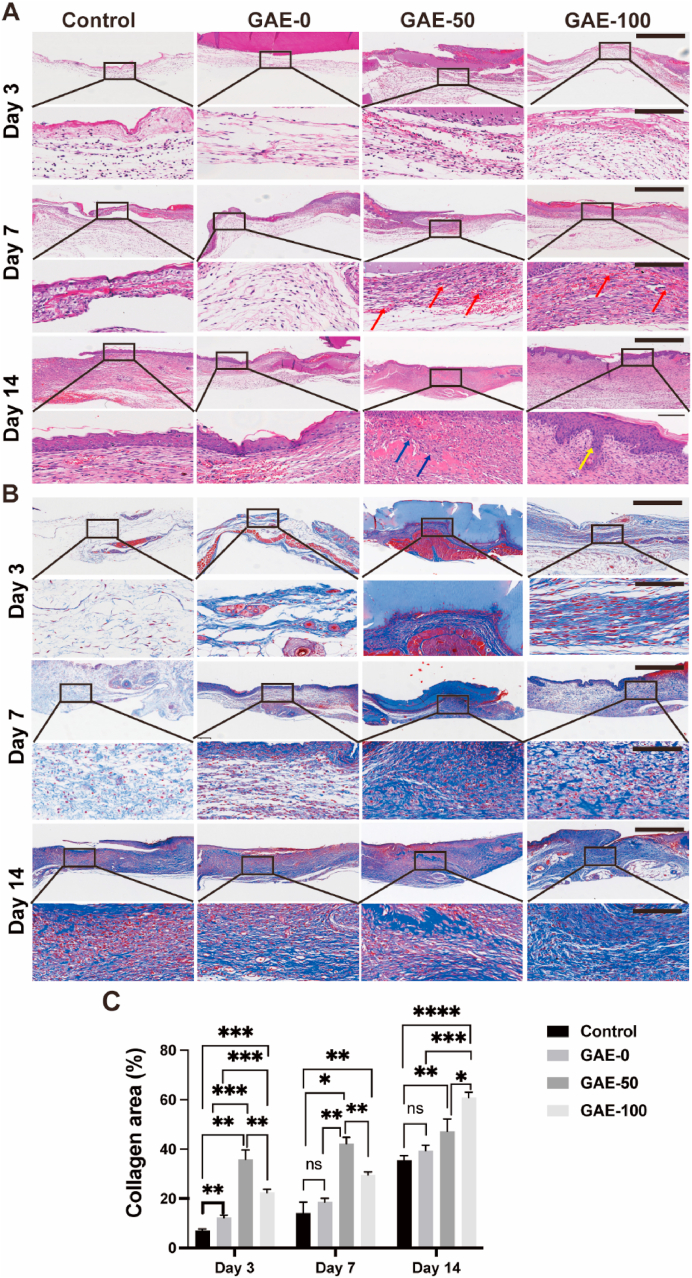
**Histological staining and histomorphological analysis of collagen formation in the wound after elastin-based hydrogels treatment. (A)** HE staining of the wound tissue after the treatment of elastin-based hydrogels for 3, 7 and 14 days. Red arrows: blood vessel. Blue arrows: collagen. Yellow arrows: hair follicle. Scale bar: 625 ​μm (top), 100 ​μm (bottom). **(B)** Masson's trichrome staining of wound tissue after the treatment of elastin-based hydrogels for 3, 7 and 14 days. Scale bar: 625 ​μm (top), 100 ​μm (bottom). **(C)** Quantitative analysis of collagen deposition after the treatment of hydrogels for 3, 7 and 14 days. Control: Wounds without hydrogel treatment. GAE-0, GAE-50 and GAE-100: Wounds treated by GelMA hydrogels, composite hydrogels containing 50% and 100% movie elastin, respectively. N ​= ​3. ∗*P* ​≤ ​0.05, ∗∗*P* ​≤ ​0.01, ∗∗∗*P* ​≤ ​0.001, ∗∗∗∗*P* ​≤ ​0.0001.

HE staining was performed to observe the histological morphology of wound tissues at different timepoints after treatment of the hydrogels (Fig. 7A). There was no conspicuous hemorrhage in all the groups. On day 3, obvious infiltration of inflammatory cells in the wound site was observed in the elastin-based hydrogels groups compared with the GAE-0 (GelMA). On day 7, plenty of micro-vessels in dermal layer could be observed in the elastin-based hydrogels groups, while the GAE-0 and control groups hydrogels showed much less angiogenesis effect. On day 14, the wound was almost healed in the hydrogels treated groups. Intact epidermis layer was developed and a thick dermis layer was also observed in all the hydrogel groups, but densely packed collagen fibers can be only observed in elastin-based hydrogels treated groups. Notably, cutaneous appendages, such as hair follicles, was regenerated in GAE-100 group, but this performance was not presented in other three groups in current study.

Masson's trichrome staining was conducted to identify collagen deposition in the wound tissue with varied treatments (Fig. 7B). The results showed that collagen accumulation increased in all the groups with time. On Day 3, only a small quantity of irregular collagen was synthesized in the control and GAE-0 groups, whereas more regular and compact collagen fibers were formed in the GAE-50 and GAE-100 groups. Similar trends could also be found on Day 7 and Day 14. It was also noticeable that thicker and denser packed collagen deposition can be observed in the GAE-50 and GAE-100 groups at the late stage of wound healing compared to other groups.

Quantitative analysis results showed that GAE-50 group outperformed the rest groups in terms of collagen area on day 3 and day 7 post-injury (Fig. 7C). Both the GAE-0 and GAE-100 groups exhibited higher collagen area than the control on day 3. The GAE-100 group displayed remarkable collagen deposition than the GAE-0 on day 7. Furthermore, this group exhibited superior effect to promote collagen formation on day 14 to the rest groups. The GAE-50 group outperformed the control group on day 14, though no significant difference compared to the GAE-0 group.

### 3.8 Elastin-based hydrogels promoted recruitment and infiltration of neutrophil and macrophages

**Fig. 8:**
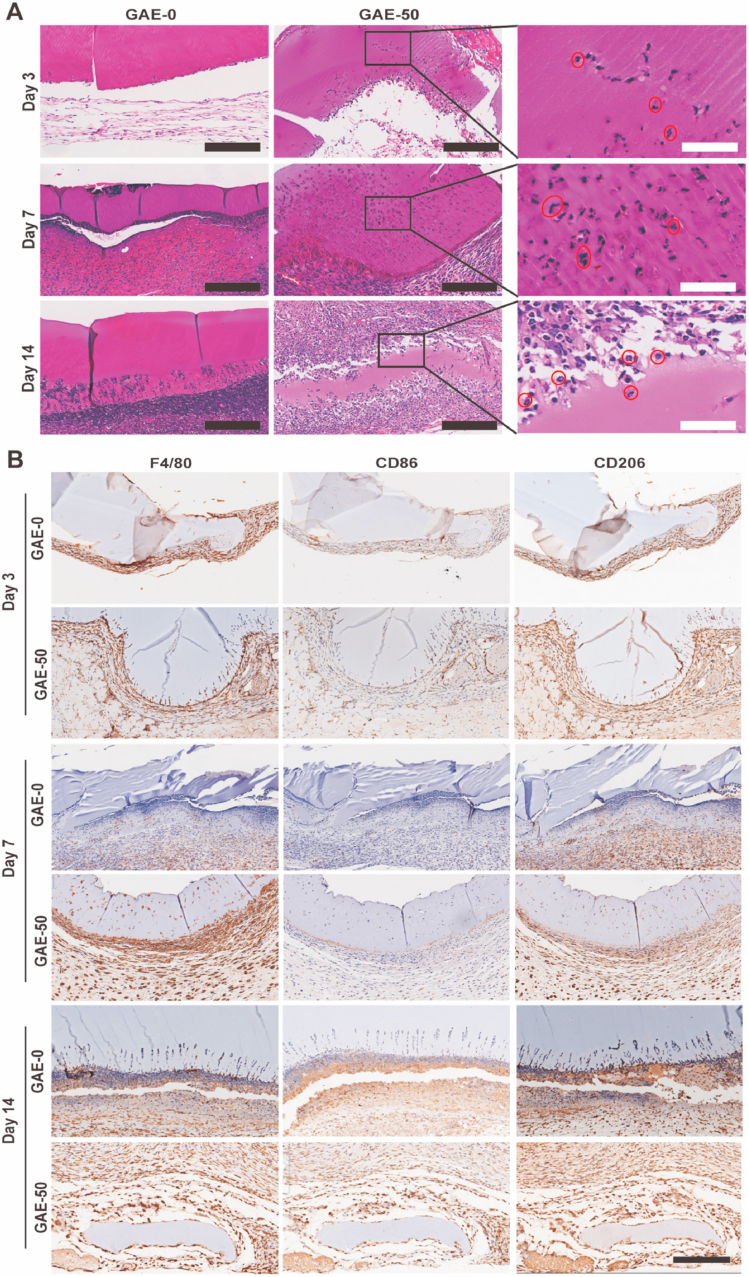
**The recruitment and infiltration of neutrophils and macrophages to the wound sites and elastin-based hydrogels at different timepoints. (A)** HE staining of wound tissue after treatment of elastin-based hydrogels for 3, 7 and 14 days. The enlarged images of GAE-50 clearly showed the polymorphonuclear neutrophils, which were pointed out by the red-circles. Scale bar: 200 ​μm (Black), 50 ​μm (White); **(B)** Immunohistochemical staining of macrophages in the wound tissue after treatment of elastin-based hydrogels for 3, 7 and 14 days. F4/80: pan-macrophages; CD86: M1 macrophages; CD206: M2 macrophages. GAE-0, GAE-50, GAE-100: Wounds treated by GelMA hydrogels, composite hydrogels containing 50% and 100% modified elastin, respectively. Scale bar: 200 ​μm.

The HE staining showed that neutrophils were recruited to the hydrogels treated wounds (Fig. 8A). It was very interesting to find that neutrophils only infiltrated into the elastin-based hydrogels but not the GelMA hydrogels. In the following, GAE-50 was employed as a model to show the dynamic process of neutrophils and macrophages infiltration. The enlarged images of GAE-50 clearly confirmed the polymorphonuclear neutrophils (Fig. 8A). On day 3, some neutrophils already infiltrated into the GAE-50 and mainly existed in the area near the host tissue. After 7 days, large number of neutrophils infiltrated into the GAE-50 and distributed in all the corners of the hydrogels. On day 14, GAE-50 was almost degraded and its residual was surrounded by large amount of neutrophils.

The recruitment and infiltration of macrophages to the wound site and into the hydrogels were explored by immunohistochemistry staining (Fig. 8B), namely the F4/80 (pan-macrophages), CD86 (M1 macrophages) and CD206 (M2 macrophages). The F4/80 staining results confirmed that abundant macrophages were recruited to the wound sites treated by hydrogels since day 3. From day 3 on, a few macrophages began to infiltrate into the GAE-50, this phenomenon was not observed in the GAE-0. On day 7, the macrophages already infiltrated into the whole GAE-50. Abundant macrophages were recruited in the interface of the GAE-50 and the host tissue. In contrast, there were much less macrophages observed in the GAE-0 group compared to the GAE-50 group, and those macrophages in the GAE-0 were not located in the interface of the hydrogels and the host tissue. On day 14, the GAE-0 was partially degraded from the edge. But the GAE-50 were nearly disappeared and abundant macrophages resided around the residuals. Regarding the macrophages subtypes (M1 and M2), it was clearly observed that the macrophages migrated to the wound area treated by GAE-0 and GAE-50 were dominant CD206 positive macrophages (the anti-inflammatory M2 phenotype). But the elastin-based hydrogels group induced larger amount of M2 macrophages recruitment in the wound site to the GelMA group on both day 7 and day 14.

### 3.9 Elastin-based hydrogels facilitated macrophages activation of both M1 and M2

**Fig. 9:**
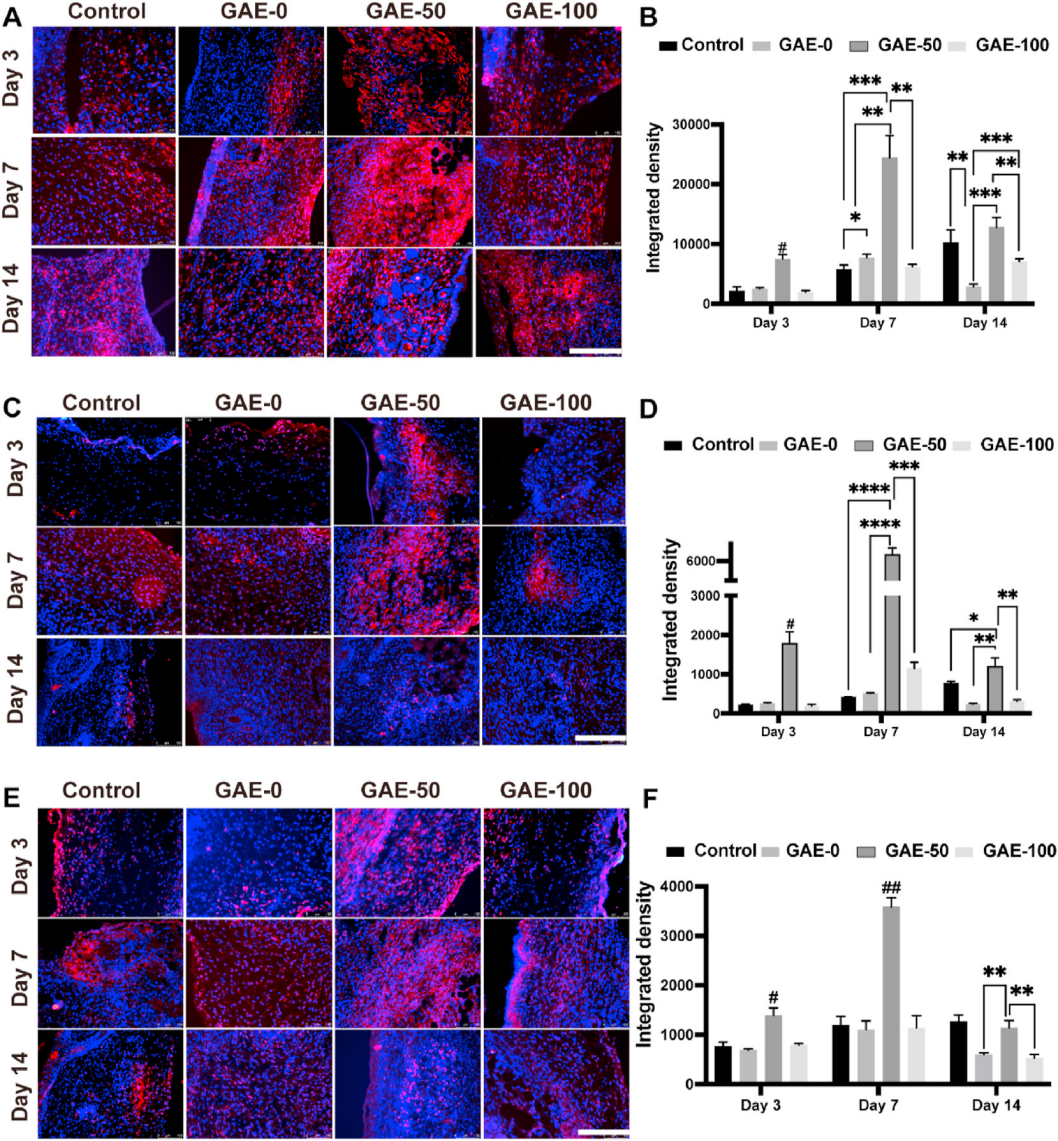
**Immunofluorescence staining of macrophages in the wound after elastin-based hydrogels treatment for 3, 7 and 14 days. (A, C, E)** Immunofluorescence staining of F4/80 (**A**), CD206 (**C**) and CD86 (**E**) in the wound tissue after treatment of the elastin-based hydrogels for 3, 7 and 14 days. F4/80: pan-macrophages; CD206: M2 macrophages; CD86: M1 macrophages. Scale bar: 200 ​μm. **(B, D, F)** Quantitative analysis of immunofluorescence staining in **A, C and E**, respectively. Control: Wounds without hydrogel treatment. GAE-0, GAE-50, GAE-100: Wounds treated by GelMA hydrogels, composite hydrogels containing 50 and 100% modified elastin, respectively. N ​= ​3. ∗*P* ​≤ ​0.05, ∗∗*P* ​≤ ​0.01, ∗∗∗*P* ​≤ ​0.001, ∗∗∗∗*P* ​≤ ​0.0001. ^#^*P* ​≤ ​0.01, ^##^*P* ​≤ ​0.001, in comparison with the other three groups at the same timepoint.

To evaluate the recruitment of varied subtypes of macrophages in the wounds after various treatments at different timepoints, immunofluorescent staining was carried out and subsequently quantified (Fig. 9). GAE-50 exhibited superior expression of F4/80 to the rest three groups on day 3 and day 7 day, and to GAE-0 and GAE-100 on day 14 (Fig. 9A and B). GAE-100 outdid GAE-0 in F4/80 expression on day 14. Additionally, GAE-50 showed dominant expression of CD206 compared to the rest groups for all the timepoints (Figue 9C, D). GAE-100 displayed higher CD206 expression than GAE-0 on day 7. Moreover, GAE-50 presented superior expression of CD86 compared to the rest groups on day 3 and day 7, and exceeded GAE-0 and GAE-100 on day 14 (Fig. 9E and F).

### 3.10 Elastin-based hydrogels promoted angiogenesis *in vivo*

**Fig. 10:**
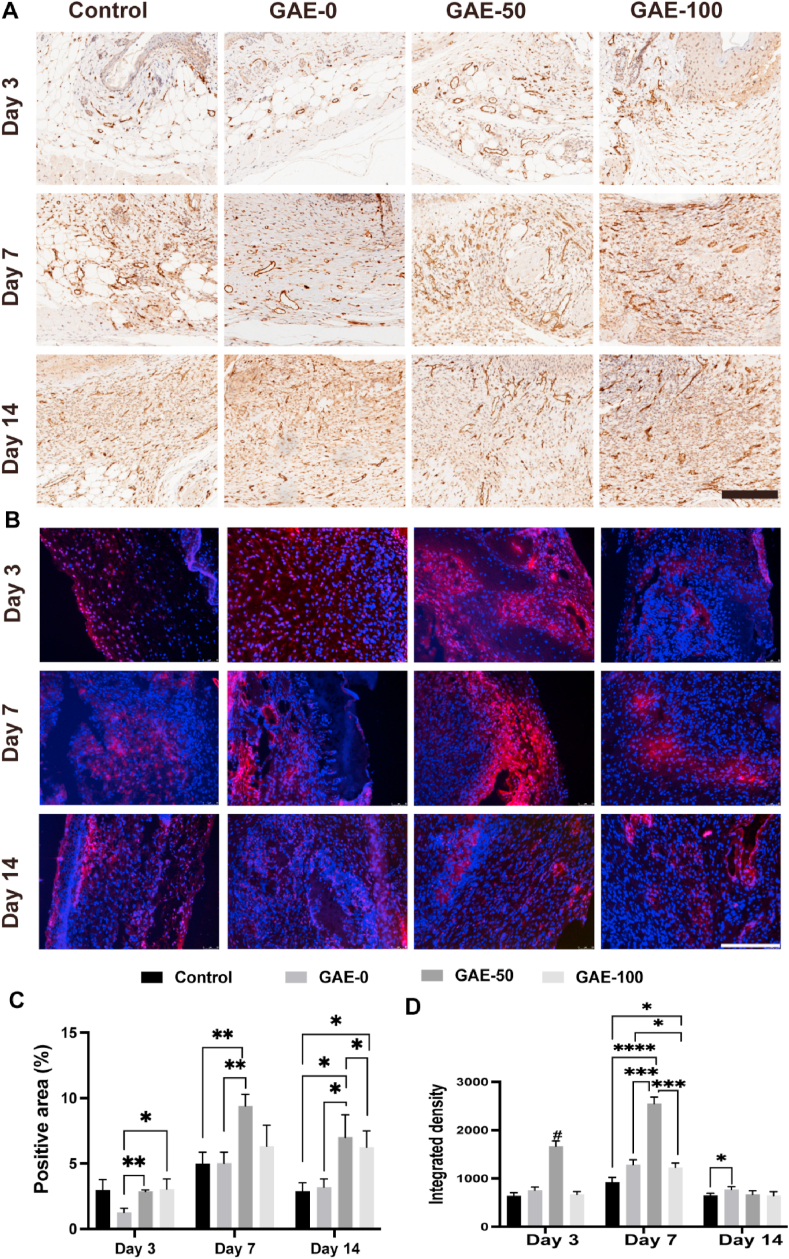
**The proangiogenic effect of elastin-based hydrogels after implantation in the wound for 3, 7 and 14 days. (A)** Immunohistochemical staining of CD31 after treatment of the elastin-based hydrogels for 3, 7 and 14 days. **(B)** Immunofluorescence staining of CD31 after treatment of the elastin-based hydrogels for 3, 7 and 14 days. Scale bar: 200 ​μm. **(C)** Quantitative analysis of immunohistochemical **(A)** and Immunofluorescence **(B)** staining. Control: Wounds without hydrogel treatment. GAE-0, GAE-50, GAE-100: Wounds treated by GelMA hydrogels, composite hydrogels containing 50 and 100% modified elastin, respectively. N ​= ​3. ∗*P* ​≤ ​0.05, ∗∗*P* ​≤ ​0.01, ∗∗∗*P* ​≤ ​0.001, ∗∗∗∗*P* ​≤ ​0.0001. ^#^*P* ​≤ ​0.001 in comparison with other three groups at the same timepoint.

Immunohistochemical and immunofluorescent staining of CD31 (marker of endothelial cell) on wound tissues were performed and quantified to evaluate the vascularization conditions in each group at different timepoints (Fig. 10). Fig. 10A showed that vessels were formed in all the groups and the amounts of vessels increased from day 3 to day 7 in all the group. On day 3, the GAE-50 outperformed the GAE-0 group in CD31 expression, but the difference was not big (Fig. 10A, C). Abundant vessels formation could be observed in GAE-50 group on day 7 and 14, this group also showed the largest percentage of CD31 positive area than the rest groups (Fig. 10A, C). The GAE-100 group also exhibited greater number of vessels than the GAE-0 and control group on day 14 (Fig. 10C). The immunofluorescent staining and quantification results further confirmed that GAE-50 group exhibited stronger fluorescence intensity compared with the rest three groups on day 3 and day 7 (Fig. 10B, D).

## 4 Discussion

Inflammation and angiogenesis are extremely critical in wound healing. The innate immune cells recruited to the wound site, particular the neutrophils and macrophages, not only play roles in the clearance of pathogens, but also produce proangiogenic growth factors and cytokines to accelerate angiogenesis and attract regenerative cells to the injury site [1,2]. Thus, biomaterials which could intrinsically recruit and modulate these innate immune cells are biological active and highly desirable for wound healing. Elastin derived peptides (EDPs) had showed chemotaxis to innate immune cells, such as neutrophils, monocytes and macrophages [18]. Additionally, recent studies reported that elastin-like recombinamer (ELR) hydrogels could recruit and modulate macrophages to facilitate tissue regeneration [23,31]. But the detailed interactions and mechanisms of elastin and innate immune cells in wound healing have not been fully elucidated. To address this, visible-light cross-linked elastin-based hydrogels were developed in this study. The *in vitro* studies confirmed that EDP-conditioned medium derived from the co-culture of these hydrogels and neutrophils significantly increased the mRNA expressions of both M1 and M2 markers in macrophages. Furthermore, the EDP-conditioned medium remarkably promoted tube formation of HUVECs in Matrigel mode. In *vivo* mice wound model, these hydrogels attracted abundant neutrophils and macrophages to the wound site and allowed the infiltration of these cells into the hydrogels, thus they acted as a reservoir of proangiogenic factors to release EDPs degraded by the infiltrated immune cells to further improve angiogenesis and attract more innate immune cells. These interactions between the elastin-based hydrogels and the immune cells generate a positive feedback loop which results in promising wound healing outcomes in current study, namely improved angiogenesis, wound contraction and dermal regeneration. The *in vitro* and *in vivo* results were in good agreement and synergistically elucidate the mechanism on how the interactions of the innate immune cells and elastin-based hydrogels can accelerate wound healing. These results are highly desirable and attractive for wound healing. Therefore, these immunomodulatory and proangiogenic elastin-based hydrogels represent promising candidate for wound healing, either as wound dressing or regenerative template.

Elastin-based biomaterials had been applied in wound healing previously [22,28]. For example, Matriderm which is porous membrane composed of collagen and hydrolyzed bovine elastin has been applied for wound healing in clinics [28]. Tropoelastin wound healing template has also been developed and showed excellent wound healing outcomes [22]. Hydrogels are suitable wound dressing materials as they can maintain a humid microenvironment in the wound site, absorb the wound exudate and protect the wound from infection [7]. Specifically, *in-situ* formed hydrogels are in high demand in wound healing, as they can fit the irregular wound site or be used as minimally invasive system [11,14]. Thus, elastin-based hydrogels have been attracting increasing interests in regenerative medicine, including wound healing [9,23,30,31]. On the other hand, hydrogels containing key components of dermal ECM, such as collagen or its degraded product gelatin, are also idea candidates for wound healing. For example, gelatin hydrogels have been explored for epidermal regeneration [29]. In the purpose of combining the merits of both elastin and gelatin, and generate a dermal ECM-mimicking microenvironment, elastin/gelatin composite hydrogels have been developed for wound healing [9,30]. While the cross-talk of elastin-based biomaterials and innate immune cells in wound healing has not been fully elucidated, we developed *in-situ* formed elastin/gelatin composite hydrogels composed of hydrolyzed bovine elastin via visible-light crosslinking in current study. ECM-derived elastin was used in our study due to its good access. This composite hydrogels present several improvements compared to previous pioneer study, in terms of biomaterials development [30]. Firstly, the plain elastin was chemically modified with AC-PEG-NHS via one-step fast reaction to introduce the acrylate groups. Furthermore, the hydrogels were formed via blue-light which can minimize the damage of the cross-linking process brought to the wound site or laden cells/bioactive factors in the hydrogels [9].

In order to develop the injectable elastin-based hydrogels, soluble elastin was modified with AC-PEG-NHS at first. The efficiency of the modification on plain elastin is related to the nucleophilicity of the amino group. When pH ​> ​pKa, the existing form of the amino group is –NH_2_ which owns stronger nucleophilicity and is conducive to the reaction. AC-PEG-NHS principally reacts with the α-amino and N-terminal amino within the pKa range of 7.6–8 [37]. Therefore, the pH of the reaction environment was settled to be above 8 to promote the modification. The side reactions, including AC-PEG-NHS reacting with sulfhydryl and hydroxyl groups of the elastin, create unstable chemical bonds, which are likely to hydrolysis and exchange with the adjacent amino groups to form amide bond [38]; and the competitive hydrolysis of AC-PEG-NHS in the alkaline solution [39]. To reduce these side reactions, 0.06 ​M NaH_2_PO_4_ buffer was employed as the buffer solution, which allowed the achievement of a high modification rate.

Mechanical property is an important aspect for the design of skin wound dressing hydrogels, as it is a key factor in determining the cell morphology, adhesion, spreading and tissue ingrowth [40]. It is also the main consideration aspect for optimization of the hydrogel formulation in this study. Previously, Holt et al. reported analyzed the skin storage modulus under a range of physiologically relevant frequencies (0.628–75.39 ​rad/s at 37 ​°C), and found that the elastic (G′) of whole skin and dermal ranged from 325.0 ​± ​93.7 ​Pa to 1227.9 ​± ​498.8 ​Pa, and 434.9 ​± ​122.1 ​Pa to 6620.0 ​± ​849.5 ​Pa, respectively [41]. It should be highlighted that the storage modulus of the developed elastin-based hydrogels ranged from around 200 ​Pa to 3000 ​kPa and was perfectly within the modulus range of the human skin. The mechanical properties of the developed hydrogels can be adjusted easily by varying the ratios of elastin. Therefore, these elastin-based hydrogels may provide matchable mechanical microenvironment to support wound regeneration. Since GAE0, GAE-20 and GAE-50 presented similar modulus, they were selected for the rest *in vitro* evaluations to study the influence of varied elastin ratio on the other physicochemical and biological properties of these hydrogels.

All the selected GAE-0, GAE-20 and GAE-50 groups displayed advantageous physicochemical properties for tissue regeneration and wound healing besides their desirable modulus, such as the porous structure, high swelling ratios and biostability. At first, the porous structure in the hydrogels is helpful for the movement of the laden cells, ingrowth of cell or tissue, and nutrients exchange [42]. High swelling ratio is good for the absorbance of wound exudation and thus providing a relatively dry environment around the wounds. Notably, the GAE-50 with higher elastin content showed superior swelling ratio. These hydrogels also presented ionic strength response, similar to our previously reported silk-based hydrogels [43], which was due to the polyelectrolyte effect and osmotic pressure difference [44]. Additionally, the elastase degradation ratio of the hydrogels decreased as increasing the elastin content, this is because of the stronger enzymatic hydrolysis of glycine and higher content of glycine in GelMA [45]. Thus, the GAE-50 with higher content of elastin, superior swelling ratio and stability against elastase degradation may possess superior ability in the controlled release of the bioactive EDPs in the wound site during the degradation.

Cytocompatibility is a critical consideration for hydrogels applied in wound healing. In this study, the precursor of the elastin-based hydrogels showed no cytotoxicity and further promoted the growth of HFF-1. From a cellular response study reported by Bax et al., the addition of soluble elastin can promote cell proliferation, which is resulted from the relationship between elastin and numerous cell-responsive facets [46]. Tropoelastin, the precursor of elastin, were able to promote the proliferation of mesenchymal stromal cells (MSCs) as reported by Yeo et al. [47]. The interactions of dermal cells with bioactive peptide sequences of elastin or tropoelastin, such as Val-Gly-Val-Ala-Pro-Gly (VGVAPG) motif, are the driving force of these promising effects. It should be mentioned that the promotion ability of EDPs on cellular viability presented concentration-dependence, which was in good consistence with previous studies applying tropoelastin in cell attachment [48,49]. Regarding the cell attachment on hydrogels, which is critically related with the surface morphology and topography of the hydrogels, should be systematically evaluated in the next stage.

Inflammation and innate immune cells greatly affect wound healing. Neutrophils and macrophages are main innate immune cells which carry out multiple roles for wound healing. Firstly, they both take part in the clearance of pathogens or debris of the wound in the inflammation stage. Additionally, neutrophils and macrophages can secret multiple bioactive factors to help on tissue regeneration. Neutrophils recruited to the wound site can attract and activate other inflammatory cells, improve angiogenesis and the proliferation of fibroblasts via improved expression of specific proteins [6]. During the growth stage, macrophages, which present dominant M2 phenotype, produce proangiogenic growth factors to promote *de novo* vessel formation and contribute to ECM regeneration in the wound [1]. Thus, the recruitment of these innate immune cells to the wound site and harnessing their activities, are highly important for wound healing. This is also the main target for the development of immunomodulatory biomaterials [15,16]. Currently, most of the reported studies on immunomodulatory biomaterials mainly focused on the modulation of macrophages, while the role of neutrophils in biomaterial-guided tissue regeneration is highly neglected [50]. Therefore, this study attempted to explore the interactions of both neutrophils and macrophages with elastin-based biomaterials during the course of wound healing.

To achieve our goals, *in vitro* co-culture of neutrophils and the hydrogel were performed at first in the purpose of mimicking the *in vivo* degradation of the hydrogels by neutrophils, as well as the generation of EDPs [2]. Neutrophils can secret neutrophil elastases which can cleave the VGVAPG sequence in the elastin-based biomaterials, and thus EDPs would release from the hydrogels. It is interesting to find the EDP-conditioned medium can effectively activate the macrophages by triggering the expression of both M1 and M2 markers. This is not surprised, as EDPs has been reported to be able to drive macrophages toward M1 or M2 phenotype in different studies [20,31]. Dale et al. had reported that EDP can promote the formation of abdominal aortic aneurysm via promoting M1 polarization of macrophages [20]. While Ibáñez-Fonseca et al. showed that ELR hydrogels could enhance skeletal muscle healing via driving M2 polarization of macrophages [31]. Though the deep mechanism need further investigation, both M1 and M2 macrophages are highly required for specific stages of wound healing, with M1 macrophages for inflammation stage and M2 macrophages for the growth stage [16]. Through the modulation of macrophages, GAE-50 presented superior ability in promoting the expression of M2 markers to GAE-20 and GAE-0, while inferior performance in trigger the expression of M1 markers to the GAE-20. Combining its outstanding physicochemical properties, the GAE-50 was chosen for *in vivo* study.

In the mice wound model, the infiltration of the first arrived neutrophils into the elastin-based hydrogels evidenced that neutrophils could degrade the elastin in the hydrogels and result in the release of EDPs. The strong chemotaxis of EDPs released further promoted the aggregation of more neutrophils and macrophages to the wound site and infiltrated into the hydrogels [18]. Activated macrophages could produce collagenase which may further degrade the elastin hydrogels and promote the infiltration of macrophages into the hydrogels, as supported by *in vitro* enzymatic degradation result. Previous study also reported that immune cells, such as neutrophil and macrophages, could filtrate into the ELR hydrogels in a rat subcutaneous model [23]. In addition to the good consistent with the previous study, current study further showed for the first time that ECM-derived elastin-based hydrogels were able to support the infiltration of these innate immune cells in a skin wound model. Furthermore, abundant M2 macrophages were presented in the wound site or infiltrated into the elastin-based hydrogels and GAE-50 group outperformed other groups in the modulation of M2 macrophages. These results were in good agreement with the *in vitro* outcomes on macrophage modulation by EDP-conditioned medium. Since M2 macrophages can produce abundant proangiogenic factors in the wound, the excellent performance of the elastin-based hydrogels in promotion of angiogenesis, collagen deposition and dermal regeneration, could be partially attributed to their ability in recruitment or modulation of M2 macrophage. This remarkable immune cell-responding feature of the elastin-based hydrogels endow them appealing bioactive platforms for wound regeneration.

Angiogenesis is a vital step in wound healing. Elastin-based biomaterials have showed proangiogenic properties in previous studies [21]. Mithieux et al. reported the angiogenesis effect of tropoelastin in mice and pig wound models [22]. Halawani et al. showed that tropoelastin can communicate to MSCs to promote tube formation of endothelial cells [51]. Robinet et al. found that elastin-derived peptides (EDPs) could enhance angiogenesis of endothelial cells [19]. The angiogenesis capacity of the ELR hydrogels were also approved in a subcutaneous model and a severe critical limb ischemia model [23,25]. Our results were in line of these studies. At first, the EDP-condition medium greatly promoted tube formation *in vitro*. Additionally, the proangiogenic effect of elastin-based hydrogels was confirmed *in vivo*. The proangiogenic properties of elastin-based biomaterials can be attributed to the multiple roles of the EDPs. Firstly, the EDPs can attract endothelia cells to the wound site and promote their angiogenesis. Moreover, EDPs can recruit macrophages to the wound site and modulate them toward M2 subtype which resulted in the production of proangiogenic factors to further improve angiogenesis. GAE-50 group showed consistent outstanding proangiogenic effect *in vitro* and *in vivo,* which may be due to its good biostability. The formation of new blood capillary is known as a key characteristic in the proliferation stage, which promises the transport of adequate cells, nutrients and oxygen to the injured tissues [52]. Accelerated angiogenesis can help to promote the synthesis of collagen fiber in the wound site. Collagen functions as a primary structural protein in the extracellular matrix. Thus, the presence of collagen in the wound sites is an indicator of effective tissue regeneration. This study demonstrated superior collagen synthesis after the treatment with the both GAE-50 and GAE-100, validating that the role of the elastin-based hydrogels in wound healing. Hence, elastin-based hydrogels play a positive role on accelerating blood vessel regeneration in wound healing.

Though very promising and interesting results were presented, the authors admitted that there were some limitations in this study. At first, only EDP-conditioned medium forms the co-culture of neutrophils and hydrogels were prepared. Future work should be conducted to evaluate the effect of EDP-conditioned medium from the co-culture of macrophages and the hydrogels. Secondly, EDP-conditioned medium was prepared by using human neutrophils in current study, due to the difficulty to obtain enough blood from the mice without sacrifice for isolation of neutrophils. Mouse neutrophils may be used in the next step. In fact, there are a lot of further studies needed to be conducted in order to completely elucidate the underlying mechanisms between these innate immune cells and the elastin-based hydrogels.

## 5 Conclusions

In this study, dermal ECM mimicking and *in-situ* fast formed elastin-based hydrogels were successfully developed via blue-light crosslinking for wound healing application. These hydrogels presented a broad spectrum of physicochemical properties, specifically with storage moduli within the range of human skin, making them advantageous for wound regeneration. Importantly, these hydrogels displayed superior capacity in recruitment of neutrophils and macrophages to the wound site, and further modulation of macrophages toward anti-inflammatory M2 phenotype both *in vitro* and *in vivo* via releasing EDPs degraded by the activated innate immune cells. Additionally, these cytocompatible elastin-based hydrogels were able to promote angiogenesis *in vitro* and *in vivo*. Moreover, these hydrogels induced desirable collagen deposition and wound contraction in mice wound model. Given the critical role of innate immune cells, angiogenesis and collagen regeneration in wound healing, these immune-cells responding and pro-angiogenic elastin-based hydrogels can be effective regenerative platforms for wound healing. Besides their outstanding function in advancing wound healing, we foresee that these bioactive elastin-based hydrogels may be further explored as versatile platforms for *in vivo* drug and cell delivery for applications in varied fields of regenerative medicine.

## Credit author statement

**Duomei Tian**: Methodology - *in vitro* cellular and *in vivo* experiments, Data curation, Writing - original draft, review & editing. **Huanhuan Wan**: Methodology - Synthesis and characterization of hydrogels, *in vivo* experiments, Data curation, Writing - original draft, review & editing. **Jiareng Chen**: Investigation, Methodology - Modification and characterization of elastin, synthesis of hydrogels, *in vitro* cellular experiments, Data curation, Writing - original draft, review & editing. **Yongbin Ye**: Investigation, Methodology – Neutrophil co-culture experiment, *in vitro* cellular experiments, Data curation, Writing - original draft, review & editing. **Yong He**: Investigation, Formal analysis, Data curation, Writing - review & editing. **Yu Liu**: Investigation, Formal analysis, Data curation, Writing - review & editing. **Luyao Tang**: Methodology – characterization of hydrogels, Formal analysis, Data curation. **Zhongyuan He**: Methodology - *In vivo* experiments. **Kaizheng Liu**: Methodology - *In vitro* experiments. **Chongjian Gao**: Methodology - mechanical analysis experiment. **Shenglin Li:** Methodology - FTIR and SEM experiments. **Qian Xu**: Investigation, Formal analysis. **Zheng Yang**: Methodology - *In vivo* experiments, Formal analysis. **Chen Lai**: Methodology - *In vitro* experiments, SEM. **Xiaojun Xu**: Investigation, Formal analysis. **Changshun Ruan**: Investigation, Formal analysis. **Yunsheng Xu**: Conceptualization, Formal analysis, Data curation, Writing - review & editing. **Chao Zhang**: Conceptualization, Formal analysis, Data curation, Writing - review & editing. **Liang Luo**: Conceptualization, Formal analysis, Data curation, Writing - review & editing. **Leping Yan**: Conceptualization, Formal analysis, Data curation, Writing - review & editing.

## Declaration of competing interest

The authors declare that they have no known competing financial interests or personal relationships that could have appeared to influence the work reported in this paper.

## Appendix A Supplementary data

figs1 figs1

figs2 figs2
