# Supplementary figures and images for: In-situ formed elastin-based hydrogels enhance wound healing via promoting innate immune cells recruitment and angiogenesis
Source: Mater Today Bio. 2022 May 21;15:100300. doi: 10.1016/j.mtbio.2022.100300 (PMC9157562; doi:10.1016/j.mtbio.2022.100300)

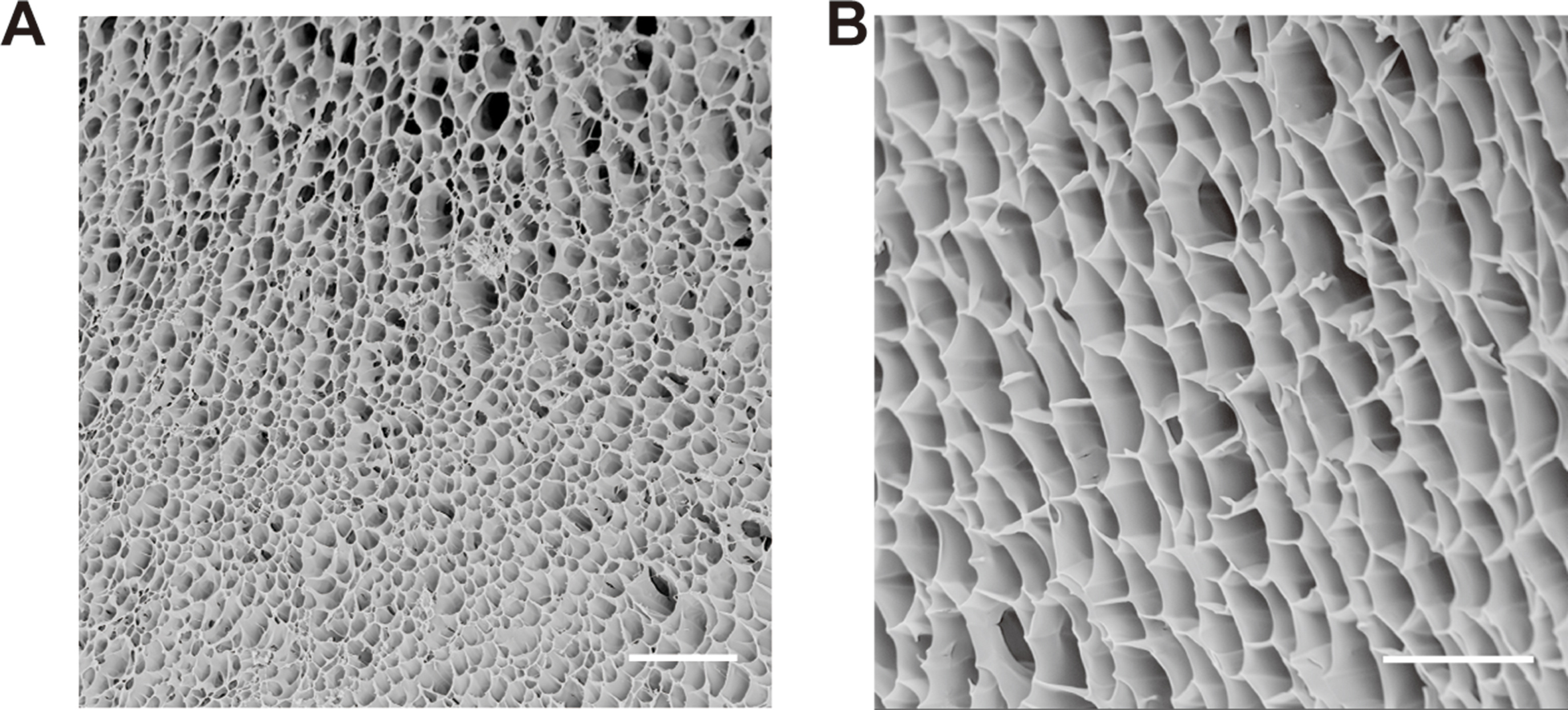

Supplement: figs1 [file figs1.jpg]

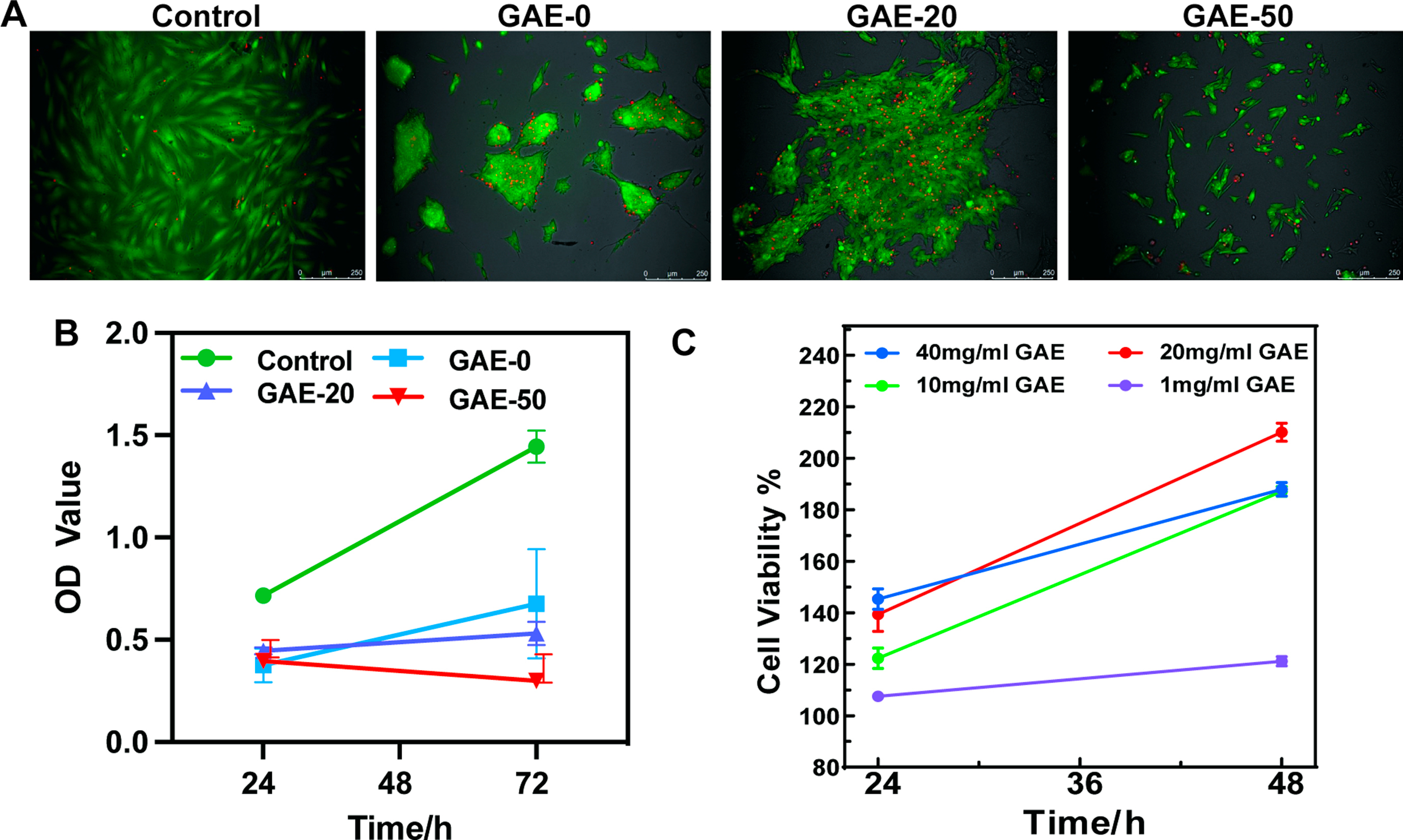

Supplement: figs2 [file figs2.jpg]
